# Structural disruption of Ntox15 nuclease effector domains by immunity proteins protects against type VI secretion system intoxication in Bacteroidales

**DOI:** 10.1128/mbio.01039-23

**Published:** 2023-06-22

**Authors:** Dustin E. Bosch, Romina Abbasian, Bishal Parajuli, S. Brook Peterson, Joseph D. Mougous

**Affiliations:** 1 Department of Pathology, Carver College of Medicine, University of Iowa, Iowa City, Iowa, USA; 2 Department of Microbiology, University of Washington School of Medicine, Seattle, Washington, USA; 3 Howard Hughes Medical Institute, University of Washington, Seattle, Washington, USA; 4 Microbial Interactions and Microbiome Center, University of Washington, Seattle, Washington, USA; Instituto Gulbenkian de Ciência, Oeiras, Portugal

**Keywords:** microbiome, Bacteroides, type VI secretion system, inflammatory bowel disease, structural biology

## Abstract

Bacteroidales use type VI secretion systems (T6SS) to competitively colonize and persist in the colon. We identify a horizontally transferred T6SS with Ntox15 family nuclease effector (Tde1) that mediates interbacterial antagonism among Bacteroidales, including several derived from a single human donor. Expression of cognate (Tdi1) or orphan immunity proteins in acquired interbacterial defense systems protects against Tde1-dependent attack. We find that immunity protein interaction induces a large effector conformational change in Tde nucleases, disrupting the active site and altering the DNA-binding site. Crystallographic snapshots of isolated Tde1, the Tde1/Tdi1 complex, and homologs from *Phocaeicola vulgatus* (Tde2/Tdi2) illustrate a conserved mechanism of immunity inserting into the central core of Tde, splitting the nuclease fold into two subdomains. The Tde/Tdi interface and immunity mechanism are distinct from all other polymorphic toxin–immunity interactions of known structure. Bacteroidales abundance has been linked to inflammatory bowel disease activity in prior studies, and we demonstrate that Tde and T6SS structural genes are each enriched in fecal metagenomes from ulcerative colitis subjects. Genetically mobile Tde1-encoding T6SS in Bacteroidales mediate competitive growth and may be involved in inflammatory bowel disease. Broad immunity is conferred by Tdi1 homologs through a fold-disrupting mechanism unique among polymorphic effector–immunity pairs of known structure.

**IMPORTANCE:**

Bacteroidales are related to inflammatory bowel disease severity and progression. We identify type VI secretion system (T6SS) nuclease effectors (Tde) which are enriched in ulcerative colitis and horizontally transferred on mobile genetic elements. Tde-encoding T6SSs mediate interbacterial competition. Orphan and cognate immunity proteins (Tdi) prevent intoxication by multiple Tde through a new mechanism among polymorphic toxin systems. Tdi inserts into the effector central core, splitting Ntox15 into two subdomains and disrupting the active site. This mechanism may allow for evolutionary diversification of the Tde/Tdi interface as observed in colicin nuclease–immunity interactions, promoting broad neutralization of Tde by orphan Tdi. Tde-dependent T6SS interbacterial antagonism may contribute to Bacteroidales diversity in the context of ulcerative colitis.

## INTRODUCTION

The Bacteroidota phylum is a major component of the healthy intestinal microbiome community. Specific taxa within this phylum, and their relative abundances have been linked to diverse diseases including components of the metabolic syndrome (1 - 3), viral infection (4), and colorectal carcinogenesis (5). Members of the Bacteroidales order may also have a role in severity and progression of inflammatory bowel disease (IBD) (6). IBD includes both Crohn’s disease (CD) and ulcerative colitis (UC), related diseases with distinct pathophysiology. Crohn’s disease is characterized by “full-thickness” inflammation extending through all layers at any location along the gastrointestinal tract. In contrast, the inflammation of UC is confined to the superficial layers of the colon. Active inflammation in Crohn’s disease correlates to *Phocaeicola vulgatus* abundance, while reduced *Bacteroides* spp. were observed in UC patients with diarrhea and rectal bleeding (7, 8). Several Bacteroidales were among the taxa with greatest fluctuation over time in a large longitudinal IBD microbiome study, suggesting dynamic re-organization of their niche(s) during disease development (9). In summary, correlative human studies suggest that alterations among specific Bacteroidaceae may contribute IBD progression, and patterns differ between Crohn’s disease and UC. The longitudinal changes in Bacteroidales abundance observed in IBD may be influenced by competitive interactions (10, 11).

Bacteroidales and other commensals in the intestinal microbiome engage in contact-dependent interbacterial antagonism using toxin secretion systems (11, 12). Type VI secretion system (T6SS) gene clusters encode at least 13 structural proteins that assemble into a needle-like apparatus for delivery of effectors (toxins) into neighboring bacteria (13). A contractile sheath composed of TssB and TssC propels an inner tubular structure composed of hexameric hemolysin co-regulatory protein (Hcp) through multi-protein baseplate and membrane complexes. Hcp and the T6SS tip structure are secreted into recipient periplasm and/or cytoplasm, carrying payloads of effectors (toxins) that promote cell death (14). T6SS^iii^ gene clusters found in Bacteroidales are distantly related to model systems in Pseudomonadota (T6SS^i^), encoding nine shared core structural proteins and five core proteins restricted to Bacteroidales (15). Three prototypic T6SS genetic architectures have been described in Bacteroidales, two found on mobile elements a third largely restricted to *Bacteroides fragilis* (15). Conjugative transfer of mobile GA1 and GA2 type T6SS among Bacteroidales has been observed within the intestinal microbiome (16, 17). *Bacteroides* spp. utilize these T6SS to antagonize non-immune Bacteroidales (12, 18) and establish and maintain colonization (11).

T6SS effector delivery frequently involves direct interaction with Hcp or the tip structure (19, 20). However, some effector domains are translationally fused to secreted core components (21, 22). For example, pyocin and colicin type DNAse domain fusions with Hcp mediate T6SS-dependent antagonism in Pseudomonadota (21). T6SS effectors employ a striking array of activities to disrupt essential biologic functions, spanning enzymatic degradation of key small molecules, post-translational modification of essential proteins, and disruption of membrane and peptidoglycan layer barriers (14). Effectors with novel toxin 15 (Ntox15) DNAse domains, also known as toxin_43 domains, degrade recipient genomic DNA (23). Ntox15 effectors in the soil bacterium *Agrobacterium tumefaciens*, T6SS DNase effectors (Tde1-2), mediate competition *in planta*. Secretion of *A. tumefaciens* Tde effectors requires loading onto the C-termini of tip structure proteins with aid of adaptor/chaperone proteins (24, 25). Loading of Tde1/2 onto the tip structure is required for efficient sheath assembly and T6SS secretion (26). Cognate immunity proteins are encoded adjacent to T6SS effectors and neutralize their activity to prevent intoxication of self and kin (27). Immunity proteins usually prevent intoxication by direct occlusion of the effector active site (28, 29). Less common mechanisms include enzymatic antagonism of effector activities, e.g., reversal of toxin-mediated ADP ribosylation (30). Arrays of immunity proteins are also found encoded by gene clusters unassociated with a T6SS apparatus, termed “orphan” immunity proteins. These AIDs are frequently on mobile genetic elements and horizontally transferred to confer protection from type VI attack, impacting competitive colonization among Bacteroidales (27).

Bacteroidales T6SS have been implicated in mouse models of infectious colitis. Commensal *B. fragilis* strains use T6SS to competitively exclude pathogenic enterotoxin-producing strains and protect against colitis (10). We hypothesize that T6SS effector-mediated competitive colonization underlies associations of Bacteroidales with IBD severity and progression (6, 8). In this study, we show that T6SS loci encoding Tde family nuclease effectors are specifically enriched in ulcerative colitis metagenomes compared to Crohn’s disease and healthy controls. We also show that immunity against Tde-mediated attack occurs by structural disruption of the effector domain, a mechanism unique among polymorphic toxin–immunity pairs of known structure.

## RESULTS

### Bacteroidales T6SS, Ntox15 domains, and immunity proteins are enriched in ulcerative colitis fecal metagenomes

**FIG 1:**
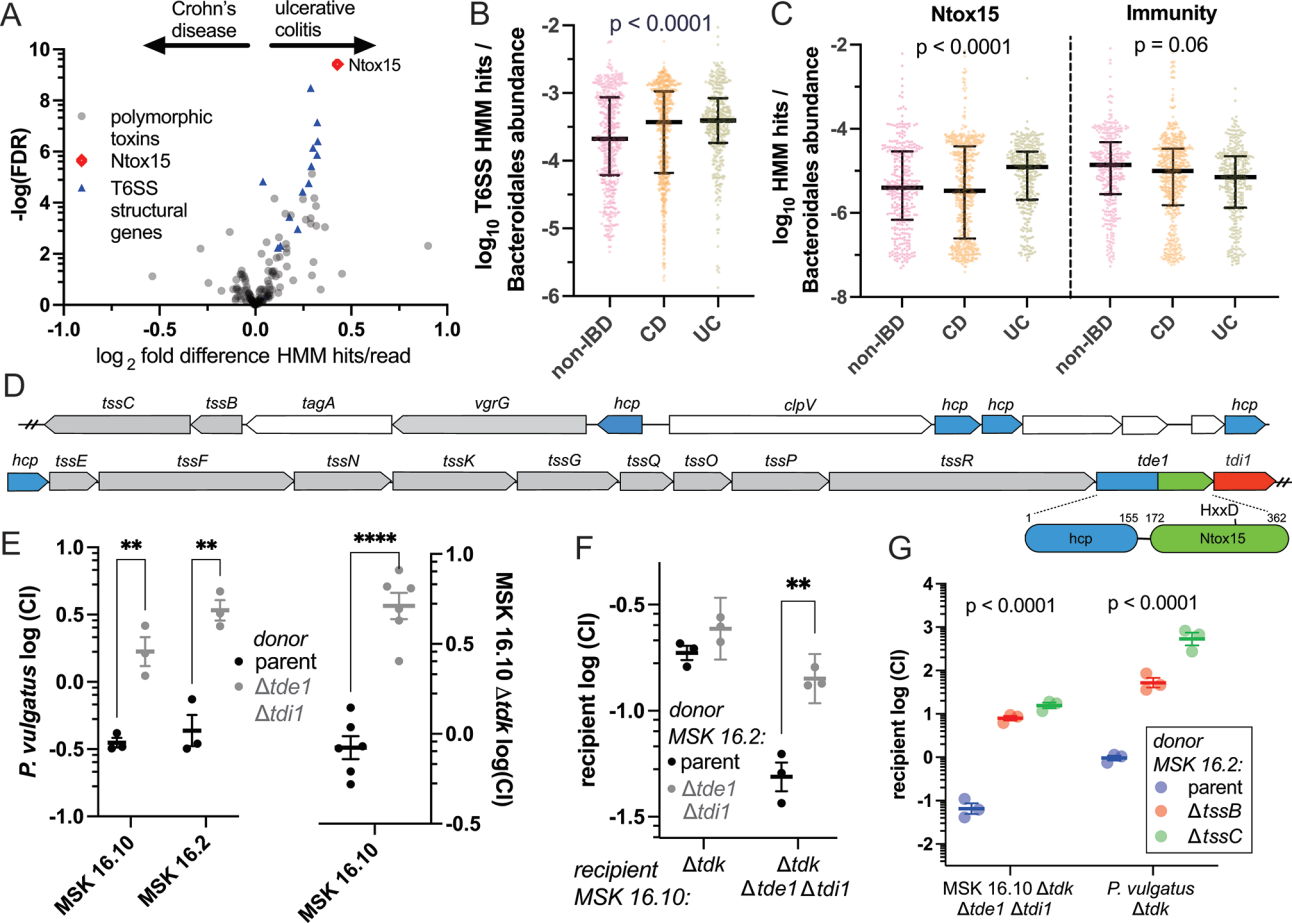
Ntox15 domains enriched in IBD metagenomes mediate T6SS-dependent interbacterial antagonism among Bacteroidales. Metagenomic sequencing reads with similarity to Bacteroidales T6SS, Ntox15 domains, and immunity proteins were detected with hidden Markov models [HMMer (33)]. (**A**) T6SS^iii^ structural genes and Ntox15 domain homologs are enriched in fecal metagenomes from patients with UC compared to CD (32). False discovery rate adjustment for multiple comparisons was with the Benjamini–Hochberg method. (**B, C**) Aggregated T6SS structural genes and Ntox15 homologs, but not the associated immunity are enriched in UC over CD and non-IBD controls after correction for relative Bacteroidales abundance. *P*-value reflects Kruskal–Wallis test. (**D**) A gene structure diagram of a T6SS-encoding locus that is identical in several genetically diverse Bacteroidales isolates from a single human donor. In addition to other T6SS structural genes (gray), there are five *hcp* genes (blue), including one fused with a C-terminal Ntox15 domain (*tde1*, green) and an immediately adjacent immunity gene (*tdi1*, red). An HxxD motif is conserved at the putative active site, predicted to confer nuclease activity. (**E**) In competitive growth experiments with *P. vulgatus* ATCC 8482, deletion of *tde1* and *tdi1* from MSK 16.10 or MSK 16.2 confers reduced relative fitness. Effector/immunity deletion is also a competitive disadvantage relative to the isogenic parental strain. Thymidine kinase (*tdk*) is deleted to confer resistance to the selection agent floxuridine (FUdR). (**F**) *tde1/tdi1* mediate competition between MSK 16.10 and MSK 16.2, isolates from a single human host. Statistical indicators reflect Student’s *t*-test: ** *P* < 0.01, *** *P* < 0.001. (**G**) Tde1-dependent antagonism requires structural sheath proteins TssB and TssC. *P*-values reflect analysis of variance (ANOVA) tests for each recipient.

Prior studies have implicated T6SS and specific effector–immunity pairs in enterotoxigenic *B. fragilis* colitis (10). Based on these data, we asked whether T6SS^iii^ loci and particular effector types are enriched among bacterial communities of IBD patient fecal samples. We constructed hidden Markov models (HMM) for the conserved Bacteroidales T6SS structural proteins, as well as ~150 Bacteroidales polymorphic toxin domain families and associated immunity proteins (31). These HMMs were applied to a large collection of publicly available shotgun metagenomic sequencing data from humans with inflammatory bowel disease and healthy controls (32). This Integrative Human Microbiome Project cohort included biweekly stool samples from 67 subjects with Crohn’s disease, 38 with UC, and 27 non-IBD controls (9). Strong correlation of HMM hits among the T6SS structural proteins was observed, as expected, because the corresponding genes are co-inherited in T6SS loci (Fig. S1A). HMM hit quantities per reads, corrected for relative Bacteroidales abundance, of each T6SS structural protein were similar across metagenomes (Fig. S1C), except for TssH, a AAA family ATPase which was excluded from further analysis due to off-target HMM hits. T6SS structural genes were enriched in fecal metagenomes from UC patients compared to CD (Fig. 1A). The enrichment of T6SS structural gene hits in UC extends to comparison with non-IBD “healthy control” specimens and is not explained by differential relative Bacteroidales abundance (Fig. 1B). Among the ~150 polymorphic toxin domain HMMs, greatest enrichment in UC was for Ntox15 homologs (Fig. 1A). Ntox15 hits, corrected to Bacteroidales abundance were enriched in UC relative to CD and controls, while the associated immunity gene did not differ significantly across groups (Fig. 1C). There was relative enrichment of Ntox15 genes per T6SS structural gene (TssB) in ulcerative colitis samples compared to non-IBD controls, and relative depletion in Crohn’s disease (Fig. S1D; linear fit slope 1.0 [0.9–1.1] for UC, 0.5 [0.4–0.7] for non-IBD, 0.0 [-0.1–0.1] for CD). A subset of the metagenomic data analyzed were time course samples from individual subjects. Multivariate analysis indicated that T6SS hits per Bacteroidales abundance tended to increase over time in subjects with ulcerative colitis (Fig. S1B). We conclude that T6SS^iii^ and Ntox15-encoding genes are differentially abundant in the intestinal metagenomes of humans with inflammatory bowel disease, and all are enriched in UC.

### Bacteroidales from a single human intestinal community compete with T6SS encoding Tde nuclease effectors

To identify strains for functional studies on Ntox15 effectors, we queried a human intestinal commensal bacteria collection with whole genome sequencing (34). Several *Phocaeicola* and *Bacteroides* strains contain nearly identical Ntox15-encoding T6SS of the GA2 type architecture (15). These strains were all isolated from a single human donor, and their T6SS loci are encoded with neighboring mobile genetic element-related genes, highly suggestive of horizontal transfer events. Selection for specific T6SS effector and immunity pairs has importance for competitive colonization and persistence in human gut metagenomes (11). This T6SS encodes several Hcp proteins, a completely conserved (100% amino acid identity) Hcp-effector fusion with C-terminal Ntox15 domain, and an adjacent putative cognate immunity protein (Fig. 1D). This multispecies effector–immunity pair is termed Tde1 and Tdi1 to conform with nomenclature in *Agrobacterium* (23). Each genome also encodes putative effectors with rearrangement hotspot (RHS) domains adjacent to mobile element genes, which have predicted structural similarity to the Tre23 toxin of *Photorhabdus laumondii* (35).

We hypothesized that Tde1 mediates interbacterial competition among Bacteroidales. Deletion of *tde1* in two of these T6SSs, *Phocaeicola vulgatus* strains MSK 16.2 and MSK 16.10 (34), enhanced competitive survival of a recipient *P. vulgatus* strain ATCC 8482 that lacks immunity (Fig. 1E). Deletion of *tde1* and *tdi1* in MSK 16.10 also conferred a competitive disadvantage relative to the isogenic parent strain, indicating that *tdi1* likely protects against kin intoxication (Fig. 1E). The competitive disadvantage of *tde1* deletion could be explained by requirement of the Hcp domain for T6SS assembly, but the four other hcp-encoding genes may compensate. Horizontal transfer of this mobile T6SS suggested that Tde1 may mediate cell killing among strains from a single host’s microbiome. Indeed, there was *tde1*-dependent killing of MSK 16.10 by MSK 16.2 when *tde1* and *tdi1* were removed from the recipient (Fig. 1F). Antagonism of MSK16.10 by MSK 16.2 required assembly of the T6SS apparatus with sheath proteins TssB and TssC (Fig. 1G). We conclude that Hcp-Ntox15 effectors mediate T6SS-dependent competition with non-immune Bacteroidales, including strains derived from a single host.

### Bacteroidales Tde effectors are magnesium dependent DNAses with a distinct α*-*helical fold

**FIG 2:**
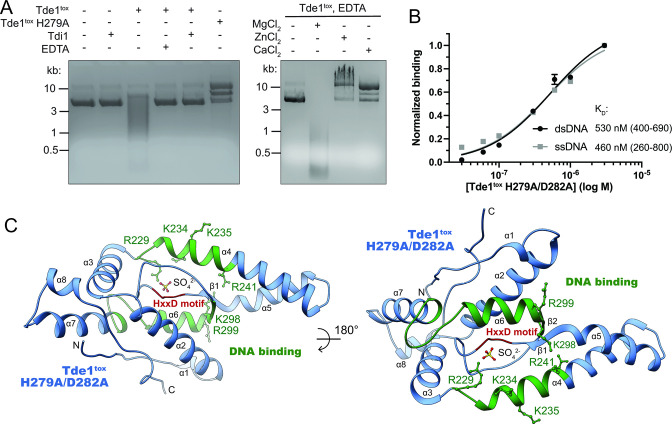
The DNAse Tde1 adopts an α*-*helical predominant fold with HxxD motif active site. (**A**) Refolded Tde1 Ntox15 domain degraded plasmid dsDNA. Nuclease activity was impaired by mutation of the HxxD motif, addition of molar excess immunity protein, or chelation of divalent cations with EDTA. Tde1^tox^ nuclease activity impairment by EDTA was reversed by addition of molar excess magnesium, but not zinc or calcium. (**B**) Tde1^tox^ with active site mutations interacted with both double- and single-stranded biotinylated oligonucleotides of random sequence, measured with biolayer interferometry. (**C**) A crystal structure of catalytically inactive Tde1^tox^ H279A/D282A domain (Table S1) was obtained by molecular replacement using an AlphaFold2 prediction (36). Tde1^tox^ adopts a single domain fold with the predicted DNA binding surface (green). Mutation of key basic residues (green sticks) to alanine or acidic residues decreased DNA binding affinity (Fig. S2F). The active site corresponds to the HxxD motif (red) and contains a modeled sulfate anion, present due to crystallization is high concentrations of ammonium sulfate.

To identify mechanisms of Ntox15 effector toxicity, we characterized the structure and enzymatic function of Tde1. The distantly homologous Ntox15 domain-containing effector Tde1 in *A. tumefaciens* exhibited DNAse activity *in vitro* and in cells (23). To examine enzymatic activity of Tde1 from *P. vulgatus,* we co-produced the Ntox15 domain (Tde1^tox^) in *E. coli* with Tdi1 to circumvent toxicity, separated it from immunity under denaturing conditions, and refolded it. Tde1^tox^ exhibited DNAse activity on plasmid dsDNA, which was abrogated by mutation of the HxxD active site (H279A) and strongly inhibited by the presence of Tdi1 or chelation of divalent cations using EDTA (Fig. 2A). EDTA-mediated inhibition was reversed by addition of molar excess magnesium salts, but not other divalent cations (Fig. 2A). Slower migration of plasmid DNA in the presence of Tde1^tox^ H279A, excess ZnCl_2_ or CaCl_2_ suggest protein binding and/or effects on supercoiling. Consistent with the toxin exhibiting non-specific DNAse activity, catalytically inactive Tde1^tox^ H279A/D282A directly interacted with 30-nucleotide single- or double-stranded DNA oligomers of random sequence, with equilibrium binding affinities near 500 nM (Fig. 2B; Fig. S2B). DNA binding affinity may be impacted by the dual point mutations in the active site.

A structural model of Tde1^tox^ H279A/D282A Ntox15 domain was obtained by X-ray crystallography with diffraction data extending to 2.9 Å resolution (Table S1; Fig. 2C). Although no close homologs of known structure were available, phases were solved by molecular replacement using an AlphaFold2 prediction model (Fig. S2C) (36). The AlphaFold2 prediction model was very similar to the experimental crystal structure; mean Cα r.m.s.d. among the eight monomers in the asymmetric unit was 1.0 Å (Fig. S2D). The Ntox15 domain adopts a globular fold which is predominantly α-helical, forming a short α-sheet between helices 5 and 6 immediately adjacent to the active site (Fig. 2C). A structural similarity search with DALI (37) revealed no close homologs within the PDB, including known nuclease structures (Z score 6.0 and Cα r.m.s.d 4.1 over 77 residues for the top hit, two pore calcium channel PDB id 6NQ1). A cavity adjacent to the mutated HxxD motif marks the active site. A sulfate ion is modeled within the active site, likely an artifact of crystallization in high concentration of ammonium sulfate. However, it may mimic accommodation of negatively charged moieties of the DNA substrate. The DNA binding site predicted with ProNA2020 (38) maps to helices 4, 6, and 7, adjacent to the active site (Fig. 2C). Coulombic surface rendering highlights relative positive surface charge surrounding the active site pocket, consistent with favorable electrostatics for interaction with negatively charged DNA (Fig. S2E). Point mutation of basic residues at the predicted DNA binding surface decreased dsDNA binding affinity (Fig. 2C; Fig. S2F). Charge reversal substitutions had greatest impact on DNA binding, supporting likely importance of electrostatic interactions. We conclude that Ntox15 domains adopt a globular fold, distinct from other nuclease families of known structure, with a structurally well-defined active site that mediates DNAse activity.

### Orphan tdi are frequent among human intestinal commensal bacteria

**FIG 3:**
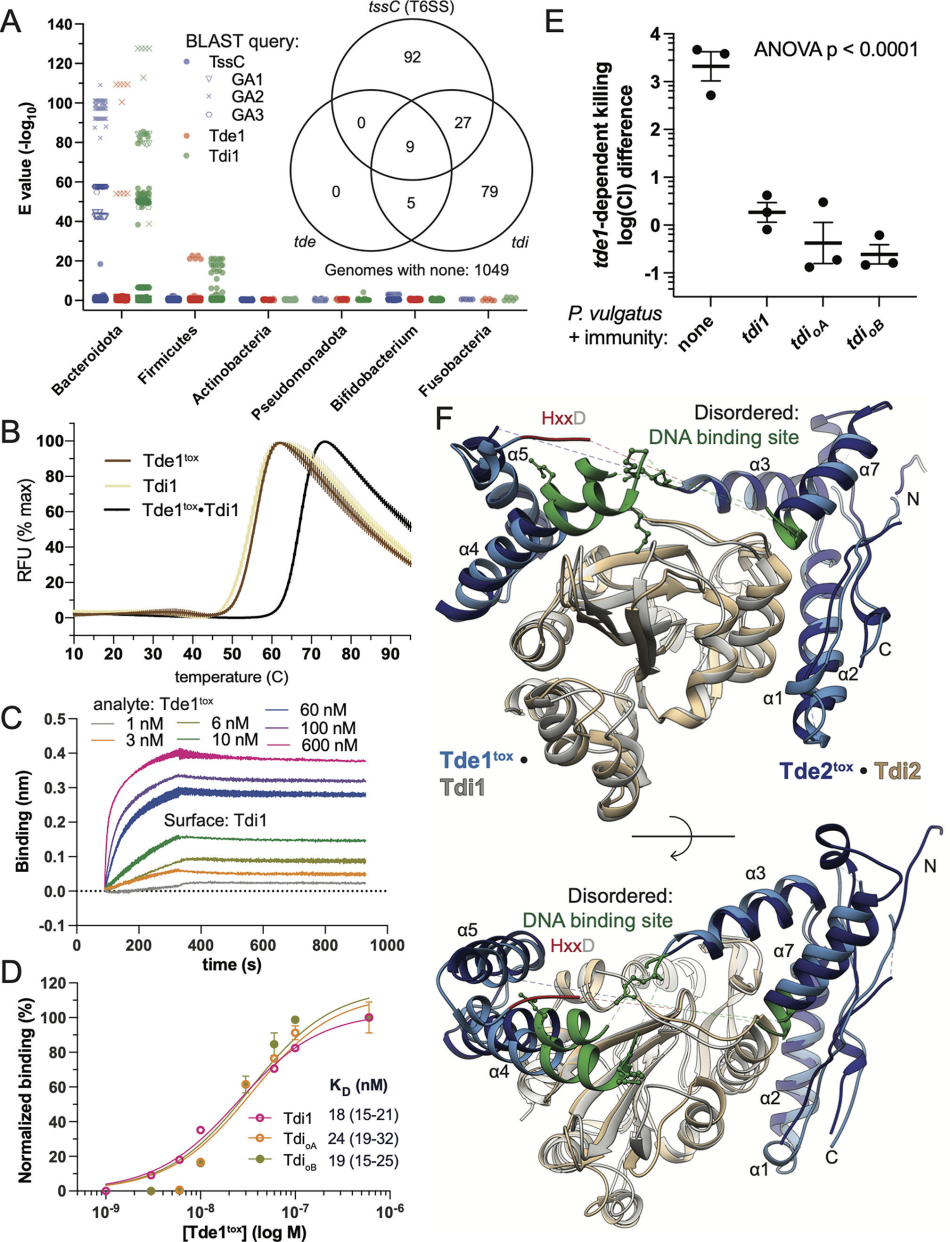
Cognate and orphan immunity proteins protect against T6SS-mediated attack by inducing a conformational shift in Tde1 to disrupt the DNA binding and active sites. (**A**) Query of Tde1, Tdi1, and representative T6SS structural protein (TssC) against a collection of ~1,200 human intestinal commensal genomes (40) with BLAST revealed predominant distribution of homologs within Bacteroidota. TssC homologs from previously described genetic architectures (GA1-3) cluster together (15). Tde1, but not Tdi1 homologs are exclusively in GA2 T6SS. Several Firmicutes harbor *tde*/*tdi* pairs not associated with T6SS. Immunity encoding genes were more abundant than *tde*. (*inset*) A Venn diagram illustrates that all identified *tde1* homologs were accompanied by *tdi. tde*/*tdi* pairs were associated with a T6SS apparatus in 9 Bacteroidota and 5 Firmicutes. However, *tdi* genes were more frequently encountered than *tde* in both phyla, indicating presence of orphan immunity genes. (**B**) Tde1^tox^ • Tdi1 exhibited higher thermal stability (melting temperature 67°C) than either component alone (55–55.5°C) in SYBR orange thermal melt experiments. (**C and D**) Biolayer interferometry demonstrated comparable equilibrium binding affinities of Tde1^tox^ for Tdi1, as well as two homologous orphan immunity proteins (K_D_ 18–24 nM). (**E**) Expression of Tdi1, as well as two orphan immunity proteins from diverse Bacteroidota protect *P. vulgatus* ATCC 8482 against *tde1*-dependent attack by *P. vulgatus* MSK 16.10. (**F**) Crystal structures of two homologous Tde^tox^ (blues) and Tdi (gray, tan) complexes demonstrate a splitting of Ntox15 into two subdomains. The subdomains are linked by the DNA binding site and the HxxD motif, which are partially disordered in the crystal structures (dotted lines). The predicted DNA binding site is green, and basic residues required for high affinity DNA interaction represented as sticks. There is high structural similarity among the homologs, indicating a conserved mode of interaction.

Ntox15 domain and core T6SS protein-encoding sequences were both enriched in UC metagenomes while immunity-encoding sequences (*tdi*) were not (Fig. 1C), raising the possibility of widespread *tdi* genes outside of T6SS loci. We assessed the distribution of T6SS, Ntox15, and immunity protein encoding genes among a large collection of human intestinal commensal genomes (34) using BLAST (39) and the Tde1-related T6SS genes as queries (Fig. 3A). The core structural *tssC* gene was identified exclusively in Bacteroidota, reflecting substantial sequence-level dissimilarity of the Bacteroidales T6SS^iii^ relative to Pseudomonadota. Ntox15 domain homologs were confidently identified (BLAST E-value < 10^−10^) in 14 Bacteroidota strains, all with GA2 T6SS architecture. In contrast, 120 strains encoded Tdi1 homologs, including all genetic architectures (Fig. 3A). Nine Bacteroidota shared a similar gene structure with the Tde1-associated system query (Fig. S3), having immediately adjacent Hcp-Ntox15 fusion and immunity proteins within the context of a GA2 T6SS structural gene cluster. More distantly related Ntox15 domain-containing proteins were encoded adjacent to Tdi1-like immunity proteins in five Firmicute genomes (*Roseburia intestinalis* and *Tyzzerella nexilis*). The genomic context and domain organization (e.g., an LXG domain fusion) suggest association with type VII secretion systems.

Notably, most of the Tdi1 homolog encoding genes were found in organisms without a Tde1 homolog, raising consideration of widespread orphan immunity among intestinal commensal bacteria (Fig. 3A) (27). The order-of-magnitude higher frequency of *tdi* compared to *tde* is consistent with the higher median frequency of *tdi* homolog sequences in metagenomes (Fig. 1C) and suggests one explanation for lack of correlation between *tdi* and disease state. Genomic context within 5 kb of these immunity genes frequently contained other putative immunity genes, distinct in sequence and domain structure, as well as genes associated with mobile genetic elements (Fig. S3). These findings suggest that Tdi1 homolog genes are frequently found in arrays of diverse immunity genes associated with mobile genetic elements, compatible with acquired immune defense (AIDs) systems (27).

### Cognate and orphan immunity proteins promiscuously engage Tde nucleases to protect against killing

Frequent occurrence of Tdi homologs in AIDs suggests that orphan immunity toward Tde toxins is an important mechanism of competition among Bacteroidales. Bacteroidales orphan immunity and effector interactions have not been biochemically characterized previously. We first characterized Tde1^tox^ H279A/D282A and Tdi1 binding with multiple biochemical platforms (Fig. 3). Tde1^tox^ interaction with Tdi1 increases thermal stability (melting temperature 67°C versus 55.5°C, Fig. 3B). A Tde1^tox^/Tdi1 dissociation constant of 18 nM was measured by biolayer interferometry (BLI, Fig. 3C and D). Two putative orphan immunity proteins were selected for further study, based on their presence in several intestinal commensal bacterial genomes, and gene structures compatible with AIDs (Fig. S3). These two proteins, termed Tdi orphan A and B (Tdi_oA_ and Tdi_oB_), share 61–65% sequence identify with Tdi1. Both orphan immunity proteins, recombinantly produced from *E. coli*, directly interacted with Tde1^tox^ H279A/D282A (Fig. 3D). Affinities of Tdi_oA_ and Tdi_oB_ for Tde1^tox^ (24 and 19 nM) were very similar to that of the cognate immunity Tdi1. Orphan immunity proteins co-expressed with the *P. vulgatus* dnLKV7 homolog Tde2^tox^ in *E. coli* also formed a stable 1:1 complex, as detected with analytical gel filtration chromatography (Fig. S4).

We next examined protective effects of orphan immunity genes in competitive growth experiments. Expression of Tdi1 from a chromosomally inserted transposon (pNBU2) in *P. vulgatus* ATCC 8482 markedly reduced *tde1*-dependent killing by MSK 16.10 (Fig. 3E). Similarly, Tdi_oA_ and Tdi_oB_ were highly protective. We conclude that orphan immunity proteins directly engage both Tde1 and Tde2 (Fig. 3B through D; Fig. S4). Orphan immunity proteins have high affinity for Tde1 and provide competitive growth advantage in co-culture with the Tde1-encoding strain *P. vulgatus* MSK 16.10.

### Immunity proteins disrupt nuclease activity by inserting into the nuclease central core: a new mechanism of polymorphic toxin immunity

**FIG 4:**
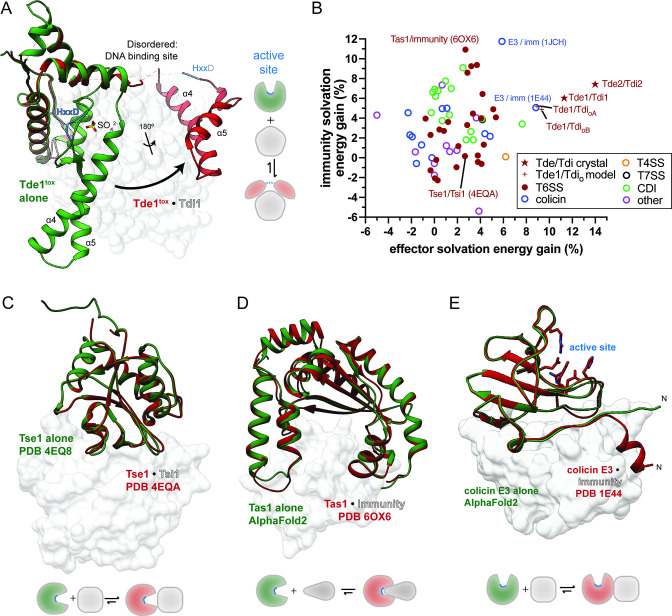
Effector fold disruption is a new immunity mechanism among polymorphic toxins. (**A**) The Tde1^tox^ alone structure (red) is superimposed on the Tde1^tox^/Tdi1 complex structure. Upon immunity binding, the split subdomains of Tde1^tox^ undergo a relative ~90° hinge motion and ~180° rotation. The DNA binding site (including helix α6) and the active site (HxxD yellow) are disrupted by the conformational shift. (**B**) Solvation energy gains of effector/immunity interface formation as percentages of monomer solvation energy were calculated with PDBePISA (41). Included structural models with PDB accession and PubMed IDs are listed in Table S2. Tde1^tox^/orphan immunity calculations are derived from comparative homology models based on the Tde1^tox^/Tdi1 structure. (**C**) The “capping” mechanism with non-disruptive steric occlusion of the effector active site is typified by the *Pseudomonas aeruginosa* T6SS-assocated peptidoglycan hydrolase Tse1/Tsi1. (**D**) Several T6SS and other polymorphic toxin/immunity interactions involve insertion of the immunity protein into a pre-formed effector active site crevice (“plugging”), typified by *P. aeruginosa* (P)ppApp synthetase Tas1/immunity. A predicted model of Tas1 alone, supported by an experimental structure of homolog RelQ (not shown, PDB 5DEC), indicates lack of large conformational shift in the effector. (**E**) A structure of colicin E3 RNAse exhibits engagement of immunity at an “exosite” separate from the enzymatic active site (42). Unlike Tde1^tox^/Tdi1, large effector conformational shifts are not predicted.

We next sought a structural explanation for how promiscuous neutralization of Tde effectors by diverse Tdi homologs is achieved. We therefore obtained crystal structures of the Tde1 Ntox15 domain in complex with Tdi1, as well as a homologous complex from *P. vulgatus* dnLKV7, Tde2^tox^ and Tdi2 (Fig. 3F). The Tde^tox^/Tdi complex homologs exhibit very similar structure despite 51% sequence identity between the Ntox15 domains, indicating a conserved mode of effector–immunity interaction. Tdi1/2 have structural homology to the Ntox15-associated immunity protein from *A. tumefaciens* (Atu4351, PDB ID 6ITW), which has been crystallized in isolation (23). Tdi1 and Atu4351 align with a Cα r.m.s.d. of 1.2 Å (Fig. S2E), although Bacteroidales Tdi1 exhibits a slightly more compact overall structure with shortening of several loops (e.g., β8-α5). When bound to immunity proteins, Tde1^tox^ and Tde2^tox^ split into two subdomains (Fig. 3F). Forty percent (17 of 43) of immunity-contacting Tde1/2^tox^ residues in the effector immunity structures form part of the central core in the globular Ntox15 domain alone structure, and many of these are highly conserved (Fig. S5). There is an ~32 amino acid region disordered in the crystal structure, corresponding to β2, α6, and the surrounding loops in the Tde1^tox^ only structure. Notably, this disordered region contains part of the HxxD active site motif and most of the DNA binding site (Fig. S5). Superposition of the Tde1^tox^ alone structure with the Tde1^tox^/Tdi1 complex indicates a conformational shift characterized by a hinge motion, as well as an ~180° relative rotation of the two Tde1^tox^ subdomains (Fig. 4A). We conclude that Tdi immunity proteins induce a marked conformational shift in Tde effectors, driving a division into two subdomains with disruption of the enzymatic active site and DNA binding motif.

Tdi1 and Tdi2 form extensive contacts with the conserved central cores of their Tde^tox^ counterparts (Fig. 3F). Upon immunity interaction, Tde effectors undergo a dramatic conformational shift, highlighted by superposition of the Tde1^tox^ alone and Tde1^tox^/Tdi1 complex structures (Fig. 4A). The immunity protein does not sterically occlude the active site, but rather splits the effector into subdomains and structurally distorts the active site, which is disordered in the crystal structures. Advances in deep learning have improved prediction accuracy for protein-protein interfaces (43), leading us to ask whether the Tde conformation shift mechanism of Tdi immunity is computationally predictable. However, AlphaFold-Multimer predicted Tde1-2^tox^/Tdi1-2 complexes inaccurately in the absence of an experimentally derived template structure (Fig. S6). The Tde^tox^α4-α5 helices interface with Tdi is approximated by the models, but effector conformational shifts and the secondary immunity interface are not identified. Thus, the Tdi1 immunity mechanism differs from previous structural investigations of T6SS-related effector–immunity pairs and cannot be reliably predicted from primary sequences with current deep learning algorithms.

To identify similar immunity mechanisms among polymorphic toxins, we compared the Tde^tox^/Tdi structure to all other polymorphic toxin–immunity pairs in the Protein Data Bank. The hydrophobic nature of Tde’s interactions with Tdi are reflected numerically in solvation energy calculations from the PDBePISA web server (41). Specifically, there is a relatively large solvation energy gain upon complex formation as compared to the Tde^tox^ monomers alone (Fig. 4B). Comparative homology models of Tde1^tox^ in complex with orphan immunity proteins, using the Tde1^tox^/Tdi1 crystal structure as a template, yielded similar solvation energy changes to the cognate immunity–effector pairs (44). As numeric markers of interface hydrophobicity, solvation energy gains were likewise calculated for each polymorphic toxin–immunity pair in the PDB (Fig. 4B). Most other effector–immunity interfaces cluster with relatively low solvation energy gains for both effector and immunity. Among the T6SS effector–immunity complexes, this pattern corresponds to immunity “capping” for steric occlusion of the effector active site, typified by the T6SS-associated Tse1/Tsi1 interaction in *P. aeruginosa* (Fig. 4C). Overlay of the Tse1 only structure (PDB 4EQ8) with the Tse1/Tsi1 complex (PDB 4EQA) demonstrates the absence of conformation shifts as found in Tde1 (Fig. 4A through C). A related mechanism of immunity, “plugging” or insertion of the immunity into a preformed effector active site cleft is illustrated with the Tas1 and immunity complex structure (PDB 6OX6) from *P. aeruginosa* (Fig. 4D). In contrast with Tde^tox^/Tdi, interactions of this type uniformly occur at the active site and do not result in large conformational shifts. While a Tas1 only structure is not available, an AlphaFold2 predicted model and structural homolog RelQ from *Bacillus subtilis* (PDB 5DEC) exhibit similar conformations to the effector in complex with immunity and an open active site crevice (Fig. 4D) (45). Several effector–immunity interactions of this pattern produced relatively high immunity solvation energy gains (Fig. 4B). The *E. coli* colicin E3 ribonuclease and immunity interfaces (PDB 1E44, 1JCH) showed parallels to Tde^tox^/Tdi1 in having relatively high effector solvation energy gain calculations (Fig. 4B) and an immunity interface that does not overlap with the effector active site (46) (Fig. 4E). Similar to other colicin nucleases, immunity is conferred by high-affinity interaction at an “exosite” (42, 47). A model of the isolated colicin E3 effector domain, predicted with AlphaFold2, shows a highly similar fold to the immunity complex, except for ~9 residues at the N-terminus. This region is predicted with low confidence in the isolated colicin E3 and assumes a short helix with extensive immunity contacts in the complex crystal structure (Fig. 4E). However, the marked conformational shift and central core interactions observed in Tde1/Tdi1 are lacking.

We conclude that Tde conformational shift and active site disruption mediated by Tdi differs from previously described polymorphic toxin–immunity interactions. Immunity contacts with the effector central core are reflected in solvation energy calculations. In contrast to the predominant active site occlusion immunity mechanisms, Tdi inserts into the Tde central core, dividing the effector domain and disrupting the active site structure.

## DISCUSSION

Our finding of essentially identical T6SS apparatus genes and Tde1–Tdi1 within diverse Bacteroidales from a single human donor is highly suggestive of recent horizontal gene transfer, possibly within the donor’s intestinal microbiome. Tde1-dependent competition among these strains implies selective pressure favoring acquisition of T6SS. Consistent with prior literature, we find T6SS gene clusters and acquired immune defense systems frequently associated with mobile genetic elements (16, 27). Active exchange and selection for genetic material relevant to T6SS-mediated attack supports previously described hypotheses that interbacterial competition among the Bacteroidota is an important determinant of the microbial community composition in individual hosts (11, 16).

Polymorphic toxins have been implicated in virulence of certain pathogenic bacteria, with mechanisms including toxin delivery to host cells (48, 49). However, disease associations with human commensal bacterial polymorphic toxins have been less thoroughly explored. In one prior study, T6SSs of commensal *B. fragilis* strains were important for competitive exclusion of pathogenic enterotoxin-producing strains (10). In this study, we find enrichment of T6SS structural genes and Ntox15 domains in patients with ulcerative colitis, suggesting positive selection for this effector immunity pair. T6SSs with *tde* homologs are found in *P. vulgatus*, and we demonstrate *tde*-mediated antagonism among three intestinally derived strains. *P. vulgatus* abundance associates with IBD disease activity (7). Furthermore, colonization with some strains of *P. vulgatus* modulates inflammation severity in rodent colitis models, although none tested in these model studies are known to encode *tde–tdi* homologs (50). Bacteroidales T6SSs and Ntox15 effectors might contribute directly to the etiology of UC, or the disease process (inflammation, epithelial disruption, etc.) may favor Bacteroidales with T6SS and *tde*. The latter hypothesis is supported by significant increases in relative T6SS gene abundance in time course metagenomic data from subjects with UC. Interestingly, UC and Crohn’s disease metagenomes exhibited opposite patterns of Ntox15 gene abundance relative to structural T6SS genes. This pattern raises the possibility that encoding Ntox15 domains may be advantageous to bacteria in UC, but detrimental in Crohn’s disease. Alternatively, there may be differential abundances of Bacteroidales with different T6SS^iii^ genetic architectures in the two disease states, which cannot be quantified with our HMM approach.

The Tde–Tdi proteins investigated in our study bear distant homology to T6SS effector–immunity pairs in *A. tumefaciens* (23). Like *A. tumefaciens* Tde1, the Bacteroidales Ntox15 domain exhibits magnesium-dependent DNAse activity. These domains are likely toxic due to non-targeted degradation of DNA in recipient cells. Given the enzymatic similarity of the effectors and the structural similarity of the immunity proteins, the immunity mechanism is very likely conserved. Mechanisms of secretion of the Bacteroidales Tde1/2 fused to Hcp are distinct from the non-covalent tip structure interactions described in *Agrobacterium* Tde1/2 (25, 26). The adaptor/chaperone proteins Tap-1 and Atu3641 required for *Agrobacterium* effector delivery are absent in Bacteroidales T6SS (25). Similarly, Bacteroidales Tde lack the N-terminal glycine zipper motif described as important for translocation of *Agrobacterium* Tde1 into recipient cells (51).

Most T6SS immunity proteins of known structure prevent intoxication of self and kin by direct steric occlusion of the effector active site (30, 52, 53), although a subset of immunity proteins also counteract effector-mediated intoxication though enzymatic activity (30). In contrast, Tdi proteins in Bacteroidales induce a large conformational change in cognate effectors, splitting the globular fold into subdomains and structurally disrupting the substrate binding and active sites. Possible mechanisms include an inherent conformational flexibility in Tde1 with selection of a two-subdomain conformation for immunity interaction, or an induced fit model of interaction where initial contacts with Tdi promote separation of the two Tde^tox^ subdomains. One possible consequence of the structural rearrangement induced in Tde could be increased efficiency of toxin destruction in the immune recipient cell. For example, Tdi insertion into the central core of Tde may facilitate proteolytic degradation of the effector.

Several parallels can be drawn between Tde/Tdi and colicin nuclease and immunity complexes. For example, colicins E3 and E9 engage immunity proteins at an “exosite” separate from the active site (54). The mechanism of immunity in these scenarios is thought to be steric and electrostatic repulsion of substrates (genomic DNA or the ribosome) (42, 55), in contrast to central core insertion and structural rearrangement of the active site seen in Tde/Tdi. Colicin nuclease immunity proteins are structurally diverse, and a prevailing hypothesis is that exosite interactions allow for evolutionary diversification at the interface, away from the conserved active site (56). Prevalent cross-reactivity of nuclease colicins and immunity proteins (55) also parallels the multi-effector interaction patterns of Tdi immunity proteins. The relatively broad specificity of Tdi immunity interactions with the central core of Tde may have evolved through exosite diversification as posited for colicin nuclease–immunity interactions. Promiscuous binding of multiple Tde by a single Tdi may be more advantageous to recipient bacteria than highly specific Tde-directed interaction (i.e., 1:1 correspondence), and may contribute to the high frequency of orphan Tdi in human commensal genome collections.

As a class of T6SS effector–immunity pairs important for competition among Bacteroidota, Tde nucleases are neutralized by unique mechanisms, including structural disruption of the active site and substrate binding surface by an immunity-induced large conformational shift. This novel immunity mechanism allows relatively broad neutralization of multiple Ntox15 domains by a single immunity protein. Further study will be required to determine how Tde and Tdi influence Bacteroidales abudance in IBD and the detailed mechanisms by which Tdi insert into the central core of Tde.

## MATERIALS AND METHODS

### T6SS gene quantitation in human intestinal metagenomes

See supplementary methods for detailed methods.

### Cloning, plasmids, and Bacteroidales genetics

See supplementary methods for detailed methods.

### Competitive growth

Bacteroidales were mixed to a final OD_600_ reading of 6.0 with 1:1 or 10:1 donor/recipient ratios and plated on BHIS with gentamycin (60 mg/mL) (57) for ~24 h at 37°C in an anerobic chamber (Anaerobe Systems, Morgan Hill, CA, USA). Bacteria were recovered in BHIS liquid media, serially diluted, and quantitatively cultured with and without 5-fluorodeoxyuridine selection. Recipient competitive indices were calculated from colony-forming units as (post-competition recipient/pre-competition recipient)/(post-competition donor/pre-competition donor). For competitive growth experiments with transposon-inserted immunity proteins, expression was induced (or mock in empty transposon controls) with anhydrotetracycline for 3 h prior to co-culture with cell–cell contact inducing conditions as above. All competitive growth experiments were performed with at least biological triplicates and at least two independently replicated experiments.

### Protein purification, crystallization, and structure determination

See supplementary methods for protein purification and crystallization methods. See Table S1 for diffraction data and refinement statistics.

### Differential scanning fluorimetry

Tde1^tox^ H279A/D282A, Tdi1, or the Tde1^tox^/Tdi1 complex were mixed at 10 µM concentration with SYPRO Orange dye at 2× concentration in X1 buffer. Temperature was increased at 0.5°C intervals every 10 s in a CFX real-time PCR detection instrument (BioRAD) with detection of dye fluorescence. Melting temperatures were assigned at the fluorescence curve inflection point. All data shown represent at least triplicate experiments.

### Biolayer interferometry

BLI experiments were conducted on an Octet Red96 instrument (Sartorius). Nucleic acid binding experiments were conducted with 30 base pair biotinylated synthetic oligonucleotides, immobilized on streptavidin biosensors. For Ntox15/immunity binding experiments, hexahistidine immunity proteins (5 mg/mL) were immobilized on NTA biosensors. Equilibrium binding dose–response curves were generated with varying concentrations of Tde1^tox^ H279A/D282A, and additional mutations thereof, in Octet kinetics buffer (Sartorius). Association and dissociation intervals were 300 and 600 s, respectively. Affinity constants were determined by one site binding curve fitting of equilibrium binding data in Prism (GraphPad) after subtraction of non-specific binding to an irrelevant surface control (biotin only). All data shown represent at least triplicate experiments.

### Nuclease activity

Plasmid DNA (2 µg of pcDNA3.1) was incubated at 37°C with Tde1^tox^ or H279A mutant (1 µM), immunity protein (10 µM), EDTA (1 mM), and/or divalent cation and chloride salts (10 mM) as indicated in a final volume of 50 µL. Reactions were halted by addition of DNA electrophoresis loading dye, and nucleic acids assessed by 1% agarose electrophoresis and ethidium bromide staining.

### Identification of T6SS, Ntox15, and immunity homologs

See supplementary methods for detailed methods.

### Structural analysis and solvation energy calculations

Polymorphic toxin and immunity protein structures were identified in the PDB using keyword searches and protein classification terms. Comparative homology models of Tde1^tox^ with Tdi_oA_ or Tdi_oB_ were constructed with SWISS-MODEL using the Tde1^tox^/Tdi1 crystal structure template (44). All structures were reviewed manually in Chimera (58) to identify effector–immunity interfaces and classify immunity mechanism. Effector and immunity solvation energy gain calculations were performed with PDBePISA (https://www.ebi.ac.uk/pdbe/pisa/) (41).

## Data Availability

Crystallographic data have been deposited to the RCSB protein data bank (accessions 8FZY, 8FZZ, and 8G0K). Metagenomic sequencing data were previously published (32) and are publicly available at the NCBI sequence read archive (BioProject PRJNA398089). Plasmids and bacterial strains generated in the study are listed in Table S3 and will be available upon reasonable request to the corresponding author.

## SUPPLEMENTAL MATERIAL

Supplemental Material mbio.01039-23-s0001.pdf Supplemental methods, Figures S1-S6, and Tables S1-S3.

ASM does not own the copyrights to Supplemental Material that may be linked to, or accessed through, an article. The authors have granted ASM a non-exclusive, world-wide license to publish the Supplemental Material files. Please contact the corresponding author directly for reuse.
