# Supplementary material for: Structural disruption of Ntox15 nuclease effector domains by immunity proteins protects against type VI secretion system intoxication in Bacteroidales
Source: mBio. 2023 Jun 22;14(4):e01039-23. doi: 10.1128/mbio.01039-23 (PMC10470768; doi:10.1128/mbio.01039-23)
Supplement: Supplemental Material — Supplemental methods, Figures S1-S6, and Tables S1-S3. [file mbio.01039-23-s0001.pdf]

Supporting Information for

Structural disruption of Ntox15 nuclease effector domains by immunity proteins protects against type VI secretion system intoxication in Bacteroidales

Dustin E Bosch[a,#], Romina Abbasian[a], Bishal Parajuli[a], S Brook Peterson[b,c], Joseph D Mougous[b,c]

[a]Department of Pathology, Carver College of Medicine, University of Iowa, Iowa City, IA, USA
[b]Department of Microbiology, University of Washington School of Medicine, Seattle, WA, USA
[c]Howard Hughes Medical Institute, University of Washington, Seattle, WA, USA
[#]Address correspondence to: Dustin E. Bosch, 340J EMRB, 500 Newton Rd, Iowa City, IA 52245
Email: dustin-bosch@uiowa.edu

This PDF file includes:

Supporting text
Figures S1 to S6
Tables S1 to S3
SI References

Extended Methods

Protein purification

Ntox15 domains, immunity proteins, and Ntox15/immunity complexes were expressed and purified from *E. coli* with a uniform strategy unless otherwise indicated. Hexahistidine-tagged protein expression from pET-Duet plasmids was induced with 1 mM IPTG for 3 hours at 37ºC in BL21 *E. coli* grown to an OD_{600} of 1.0 in LB supplemented with 1% w/v additional glucose. After lysis and centrifugation, hexahistidine-tagged proteins were isolated by NTA affinity chromatography in N1 buffer (30 mM HEPES pH 7.5, 300 mM NaCl, 30 mM imidazole), and eluted in N1 with 300 mM imidazole. Eluents were applied to a gel filtration column (Superdex 200) in X1 buffer (30 mM HEPES pH 7.5, 200 mM NaCl, 1 mM DTT). Gel filtration eluents were concentrated to 1-50 mg/mL and snap frozen for storage at -80ºC. DNA binding mutant $Tde1^{tox}$ proteins exhibited gel filtration chromatograms and Coomassie-stained SDS-PAGE gels similar to the $Tde1^{tox}$ H279A/D282A comparator.

For nuclease activity assays, hexahistidine-tagged $Tde1^{tox}$ domain was expressed and isolated in complex with untagged Tdi1 by NTA affinity chromatography as above. The $Tde1^{tox}$ /Tdi1 complex was denatured in N1 buffer with 6M guanidine HCl to remove the immunity protein. $Tde1^{tox}$ was refolded on the NTA column by linear gradient reduction of guanidine concentration, eluted in N1 with 300 mM imidazole, and further purified by gel filtration.

For NTA BLI experiments, untagged $Tde1^{tox}$ H279A/D282A domain alone was produced by cleavage of the N-terminal hexahistidine tag using tobacco etch virus protease (TEV) after isolation by NTA chromatography. A second NTA chromatography step was used to remove hexahistidine tagged TEV and uncleaved $Tde1^{tox}$, prior to gel filtration.

For crystallization of the hexahistidine tagged $Tde1^{tox}$ H279A/D282A domain alone, reductive methylation with dimethylamine-borane complex and formaldehyde was performed as described previously (1). The reductive alkylation reaction was quenched with Tris-containing buffer over gel filtration (50 mM Tris pH 8, 200 mM NaCl). The resulting N-terminal amine- and lysine-methylated $Tde1^{tox}$ H279A/D282A domain protein was concentrated to 10 mg/mL and snap frozen.

Crystals of lysine-methylated $Tde1^{tox}$ H279A/D282A domain were obtained at 5 mg/mL in X1 buffer, mixed 1:1 with crystallization solution (2 M ammonium sulfate, 200 mM sodium acetate pH 4.5). Rod shaped crystals grew to 300 × 100 × 100 µm over 5 days. Crystals were cryoprotected in crystallization solution with 25% saturated sucrose and plunged into liquid nitrogen. Diffraction data were obtained at 1.0 Å wavelength from a single crystal at 100 K temperature at the BL822 beamline (ALS, Lawrence Berkeley National Laboratory). Data were processed using HKL2000 (2). Structure solution was by molecular replacement using an AlphaFold2 (3) predicted model and Phaser (4). After placement of 8 models, the Phaser log likelihood gain was 3315 with a translation function Z-score of 42. Refinement was carried out using phenix.refine (4) interspersed with manual model revisions using the program Coot (5) and consisted of conjugate-gradient minimization and calculation of individual atomic displacement parameters; non-crystallographic symmetry restrains were applied in the initial refinement cycles.

Crystals of selenomethionine *P. vulgatus* dnLKV7 $Tde2^{tox}$ domain in complex with cognate immunity Tdi2 were obtained at 10 mg/mL in X1 buffer, mixed 1:1 with crystallization solution (15% PEG 3350 (w/v), 200 mM NaCl, and 100 mM sodium citrate pH 5.0). Crystals grew to 300 × 300 × 300 µm over 4 days. Crystals were cryoprotected in crystallization solution with 20% (v/v) ethylene glycol and plunged into liquid nitrogen. Highly redundant anomalous (SAD) data were obtained at 0.9795 Å (peak) wavelength

from a single selenomethionine crystal at 100 K temperature at the BL502 beamline (ALS, Lawrence Berkeley National Laboratory). Data were processed using HKL2000 (2). Heavy-atom searching using phenix.autosol (4) identified 26 possible sites, and refinement yielded an estimated Bayes correlation coefficient of 23.4 to 2.7 Å resolution. After density modification, the estimated Bayes correlation coefficient increased to 49.5. Approximately 70% of the selenomethionine model was constructed automatically, and the remaining portion was built manually. Refinement was carried out using phenix.refine (4) with Bijovet pairs kept separate, interspersed with manual model revisions using the program Coot (5) and consisted of conjugate-gradient minimization and calculation of individual atomic displacement parameters.

Crystals of Tde1^{tox} domain in complex with cognate immunity protein Tdi1 were obtained at 10 mg/mL in X1 buffer, mixed 1:1 with crystallization solution (15% PEG 3350, 200 mM lithium sulfate, 100 mM Bis-Tris pH 5.5). Crystals grew to 500 × 400 × 200 µm over 3 days. Crystals were cryoprotected in crystallization solution with 20% (v/v) ethylene glycol and plunged into liquid nitrogen. Diffraction data were obtained at 1.0 wavelength from a single crystal at 100 K temperature at the BL502 beamline (ALS, Lawrence Berkeley National Laboratory). Data were processed using HKL2000 (2). Structure solution was by molecular replacement using the immunity protein model from the experimentally phased *P. vulgatus* Tde2^{tox}/Tdi2 complex. After placement of 2 models, the Phaser log likelihood gain was 13824 with a translation function Z-score of 108.5. Approximately 75% of the remaining models were constructed with phenix autobuild (4). Refinement was carried out using phenix.refine (4) interspersed with manual model revisions using the program Coot (5) and consisted of conjugate-gradient minimization, calculation of individual atomic displacement parameters, and translation/libration/screw parameters (6).

Cloning, plasmids, and Bacteroidales genetics

For allelic exchange, homologous regions of 500-1000 base pairs were amplified from isolated genomic DNA (DNeasy Blood & Tissue kit, Qiagen) using PCR with high fidelity polymerase (Phusion). Ntox15 and immunity genes were synthesized with native Bacteroidales sequences and C-terminal VSVG epitope tags for transposon insertion, or codon optimization for expression in *E. coli*. Plasmids were constructed with Gibson assembly in DH5a *E. coli* (pET plasmids) or EC100D *pir* (pNBU2, pLGB30 plasmids). Mutagenesis was accomplished with an identical gene synthesis and assembly strategy, confirmed by Sanger and/or next generation sequencing.

Immunity proteins were inserted into *P. vulgatus* ATCC 8482 on the pNBU2 transposon with anhydrotetracycline inducible control as previously described (7). Briefly, immunity protein open reading frames were cloned into pNBU2_erm_P1T_DP-B1 and delivered to *P. vulgatus* by conjugation with S17-1 λpir *E. coli* (7). Insertion at *attB* sites was confirmed with PCR and Sanger sequencing. Markerless gene deletion by allelic exchange in strains MSK 16.10 and MSK 16.2 was achieved with and pLGB30 plasmids (8), confirmed with PCR and Sanger sequencing.

T6SS gene quantitation in human intestinal metagenomes

Bacteroidales protein sequences from the Tde1-Tdi1 encoding genomes were used as query sequences for BLAST to obtain homologs. Hidden Markov models were constructed from the resulting alignments using Hmmer (9). Shotgun metagenomic sequencing data and metadata corresponding to inflammatory bowel disease patients and controls included in the Human Microbiome Project (10) were obtained from the NCBI SRA (BioProject accession PRJNA389280). Relative species abundances were obtained using Metaphlan3 (11) with default settings. T6SS homolog sequences were

identified by translating DNA sequence reads into 6 possible reading frames and applying the *Bacteroides* T6SS derived hidden Markov models with Hmmer. Default settings for determination of hits were retained for simplicity and reproducibility. Data analysis was performed with HMM hit counts transformed into base 10 log scale. As expected, linear correlation between *Bacteroides* abundances and T6SS HMM hits was observed (Figure S1A), and hit numbers across T6SS structural proteins were highly correlated. To decrease the risk of detecting T6SS sequence differences due to differential Bacteroidales abundance, data were analyzed as ratios of HMM hits (log base 10) to relative Bacteroidales abundance (fraction). Data were also analyzed without correction for Bacteroidales abundance (data not shown), and similar trends were identified.

Identification of T6SS, Ntox15, and immunity homologs

Bacteroidales *hcp-Ntox15* fusions were identified in multiple genomes derived from an intestinal commensal strain collection (12). T6SS structural genes, Ntox15 domains, and immunity genes were used as query sequences to identify homologues in a larger collection of commensal genomes (13) using BLAST. T6SS genetic architecture classification was based on a previously published scheme. Genomic context, up to 10000 bp, surrounding immunity gene candidates were extracted and annotated by individual BLAST of ORFs against NCBI's nr protein database. Multiple sequence alignments were constructed with Clustal omega (14).

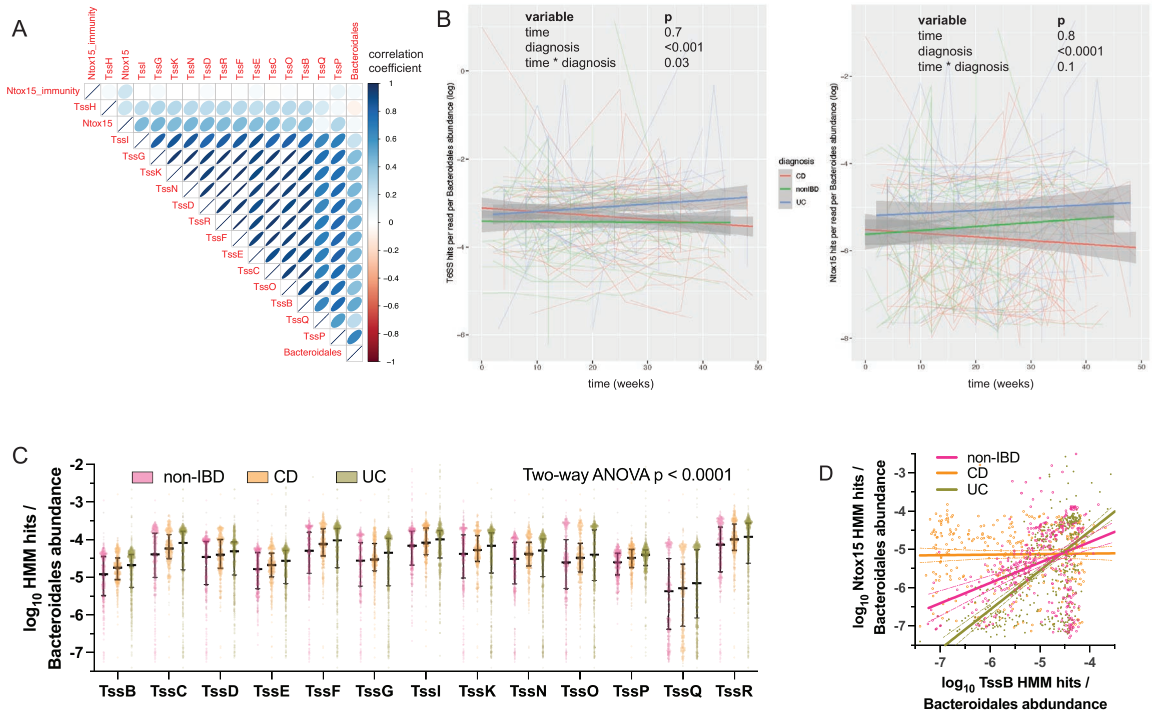

Fig. S1. Bacteroidales *T6SS structural genes are positively correlated in human intestinal metagenomes and increase with time in ulcerative colitis.* A) A correlation matrix generated with all samples used in this study highlights strong positive (blue) linear correlation of Hmmer hits per read among the T6SS structural genes in intestinal metagenomes. Ntox15 domain hits were also positively correlated to T6SS structural genes. Weak or no significant correlation was observed to Ntox15-related immunity genes or relative abundances of Bacteroidota or Bacteroidales taxa. B) A subset of subjects had time course samples available. Individual subjects are plotted as thin lines, while thicker lines with shaded confidence intervals represent linear fit of the aggregated data. T6SS structural gene Hmmer hits normalized to Bacteroidales abundance were significantly associated with ulcerative colitis, and changes over time differed by diagnosis. Ntox15 domain hits were also related to diagnosis, but time course data did not show a significant interaction. C) T6SS structural protein hits were consistently highest in samples in subjects with ulcerative colitis (UC), followed by Crohn's disease (CD), and controls without inflammatory bowel disease (non-IBD). D) In subjects with UC, Ntox15 domain hits were enriched in samples with high T6SS structural protein (TssB) hits. In contrast, Ntox15 domain hits were relatively depleted in Crohn's disease subjects with high TssB hits.

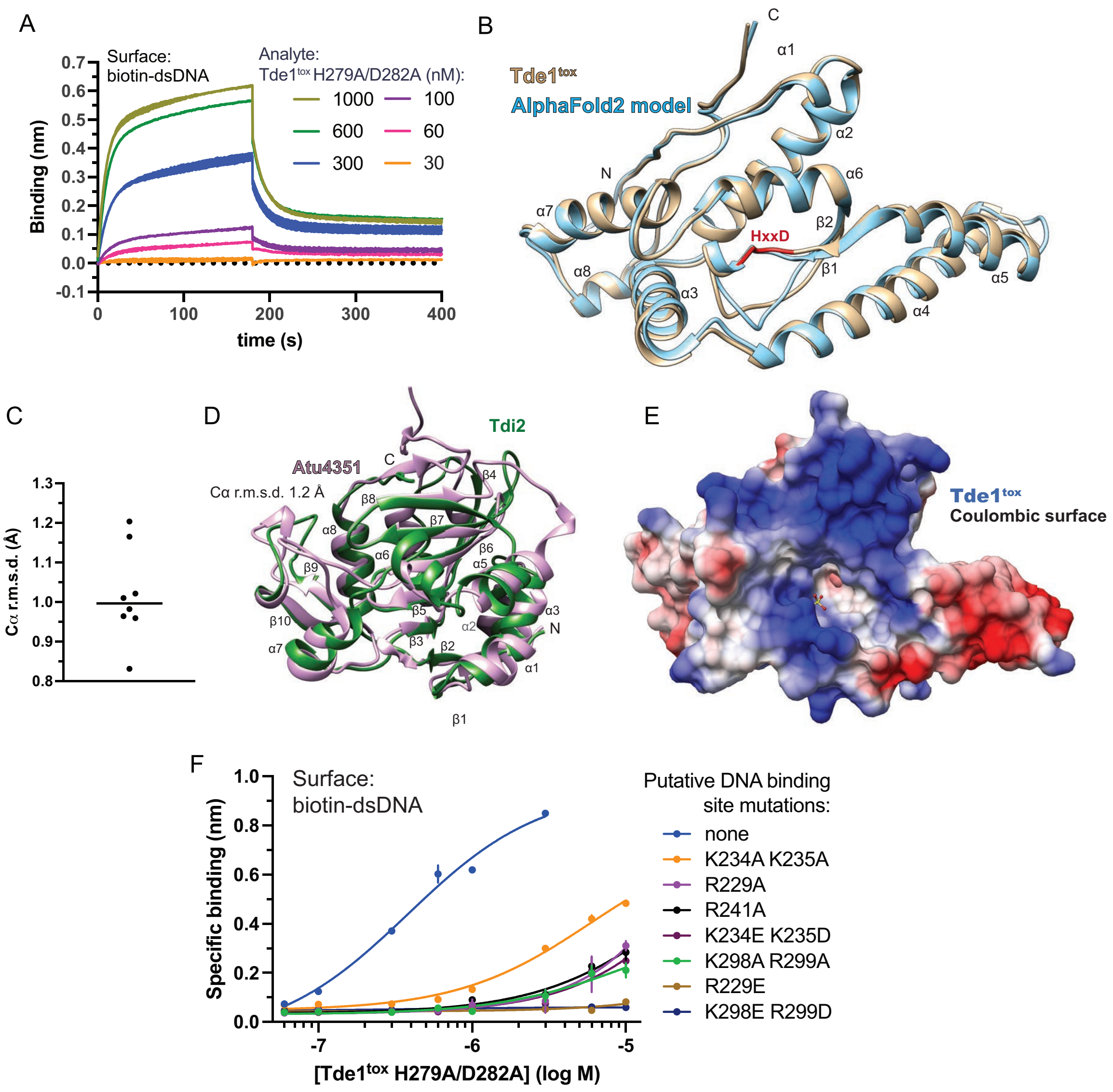

Fig. S2. *Structural features of Tdi1 and Tde1^{tox}, cofactor and substrate interactions.* A) Tde1^{tox} with active site mutations interacted with double-stranded biotinylated oligonucleotides of random sequence, measured with biolayer interferometry. B) Superposition of an AlphaFold2 model and the experimental crystallographic structure demonstrates high similarity. C) Each of the 8 monomers in the Tde1^{tox} structure were aligned to AlphaFold2 model with a mean Cα r.m.s.d. of ~1 Å. D) The crystal structure model of Tdi2 was aligned to the Ntox15-associated immunity protein from Agrobacterium tumefaciens (PDB 6ITW). The overall immunity structures were similar (Cα r.m.s.d. 1.2 Å), although several loops and secondary structure elements are shorter in the Bacteroides protein, such as β8-α5 and α6-α7. E) Coulombic surface rendering of Tde1^{tox} highlights relative positive surface charge surrounding the active site pocket, consistent with favorable electrostatics for interaction with negatively charged DNA. F) Mutation of several basic residues at the predicted DNA binding surface reduced interaction with biotinylated dsDNA in the H279A/D282A background, as measured with biolayer interferometry. Charge reversal substitutions at positions 229 and 298-299 eliminated detectable specific binding. Mutant Tde1^{tox} affinity constants could not be accurately assessed because equilibrium binding saturation was not reached at 10 μM.

A

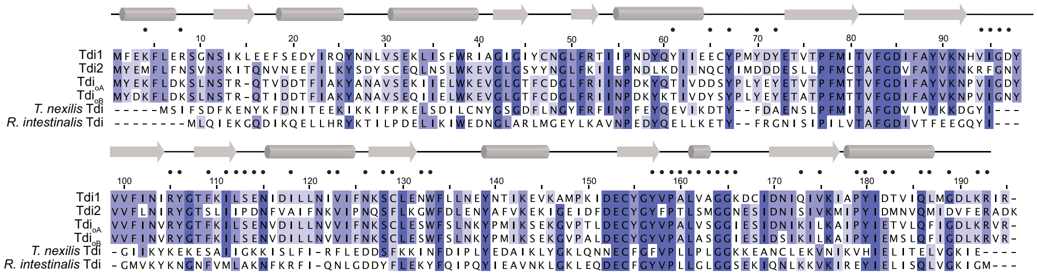

B

Designation (this study)	Species	NCBI accession	Start	End	Percent identity with native immunity: overall	Percent identity with native immunity: Ntox15 contacts
Multispecies native immunity Tdi1	*Phocaeicola vulgatus*	NZ_QSVY01000008.1	22935	23519	100	100
	Phocaeicola vulgatus	NZ_QSWA01000009.1	22941	23525		
	Bacteroides sp. 3_1_33FAA	NZ_QSVH01000017.1	45938	45354		
	Parabacteroides distasonis	NZ_QRKE01000027.1	21162	20578		
NA	*Phocaeicola vulgatus*	NZ_QRYT01000032.1	43751	43167	89	n.c.
orphan immunity 1 Tdi_{oA}	*Butyricimonas virosa*	NZ_QRZA01000073.1	1890	2471	65	77
	Bacteroides faecis	NZ_QSVL01000072.1	3413	2832		
	Parabacteroides merdae	NZ_QSII01000024.1	29826	30407		
	Parabacteroides distasonis	NZ_QSVO01000036.1	4985	5566		
	Bacteroides clarus	NZ_QRZG01000024.1	5354	5935		
	Bacteroides clarus	NZ_QRZB01000024.1	5348	5929		
	Odoribacter splanchnicus	NZ_QRYW01000026.1	15025	15606		
	Bacteroides uniformis	NZ_QRZP01000023.1	55010	55591		
	Bacteroides uniformis	NZ_QRZC01000026.1	25246	24665		
	Phocaeicola dorei	NZ_QRZL01000001.1	84103	83522		
	Bacteroides xylanisolvens	NZ_QRYV01000021.1	64668	65249		
NA	*Phocaeicola plebeius*	NZ_QRQK01000028.1	56865	56284	65	n.c.
NA	*Parabacteroides merdae*	NZ_QRPL01000038.1	3349	3933	65	n.c.
orphan immunity 2 Tdi_{oB}	*Phocaeicola plebeius*	NZ_QRML01000070.1	820	1401	61	71
	Phocaeicola plebeius	NZ_QRTR01000051.1	1925	1344		
	Phocaeicola plebeius	NZ_QRTL01000048.1	1931	1350		
	Phocaeicola plebeius	NZ_QRHQ01000003.1	27478	28059		
	Phocaeicola plebeius	NZ_QSTF01000064.1	20749	21330		
	Phocaeicola plebeius	NZ_QSQT01000049.1	19356	19937		

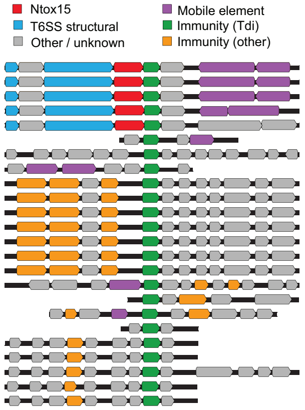

Fig. S3. *Ntox15-associated immunity proteins are associated with T6SS or acquired interbacterial defense systems in human intestinal commensal genomes.* A) Bacteroidales Tdi protein sequences are aligned with distant homologs from *Tyzzerella nexilis* and *Roseburia intestinalis*. Dots correspond to Tde1 contact residues in the Tde1/Tdi1 structure. B) Genomic context is shown for the most highly similar BLAST hits among a collection of intestinal commensal bacteria genome sequences. Several strains encode an hcp-Ntox15 domain fusion protein with cognate immunity in the context of a T6SS locus. Other Tdi proteins were frequently encountered in the context of additional annotated T6SS immunity genes and/or mobile element-related genes, compatible with AIDs, and absence of an adjacent Tde.

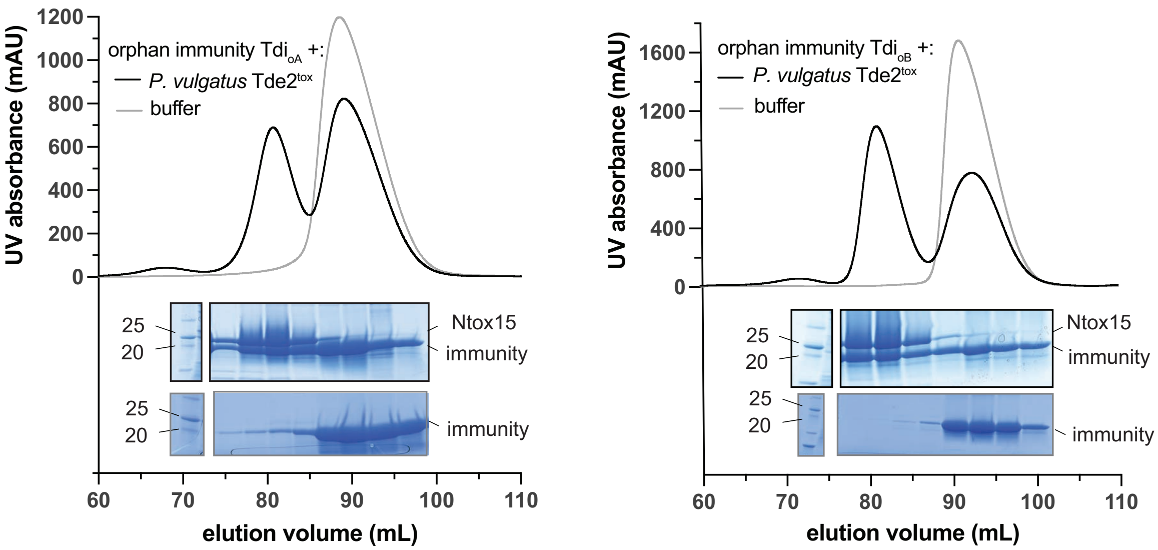

Fig. S4. *Orphan immunity proteins co-migrate with Tde2^{tox}.* Orphan immunity proteins were expressed alone or with *P. vulgatus* dnLKV7 Tde2^{tox} domain in *E. coli* and complex assembly assessed with size exclusion chromatography. Tde2^{tox} co-migrated with each orphan immunity protein, eluting at a volume compatible with 1:1 Tde^{tox} / Tdi complex molecular weight. Chromatography fractions were sampled for SDS-PAGE and Coomassie stain.

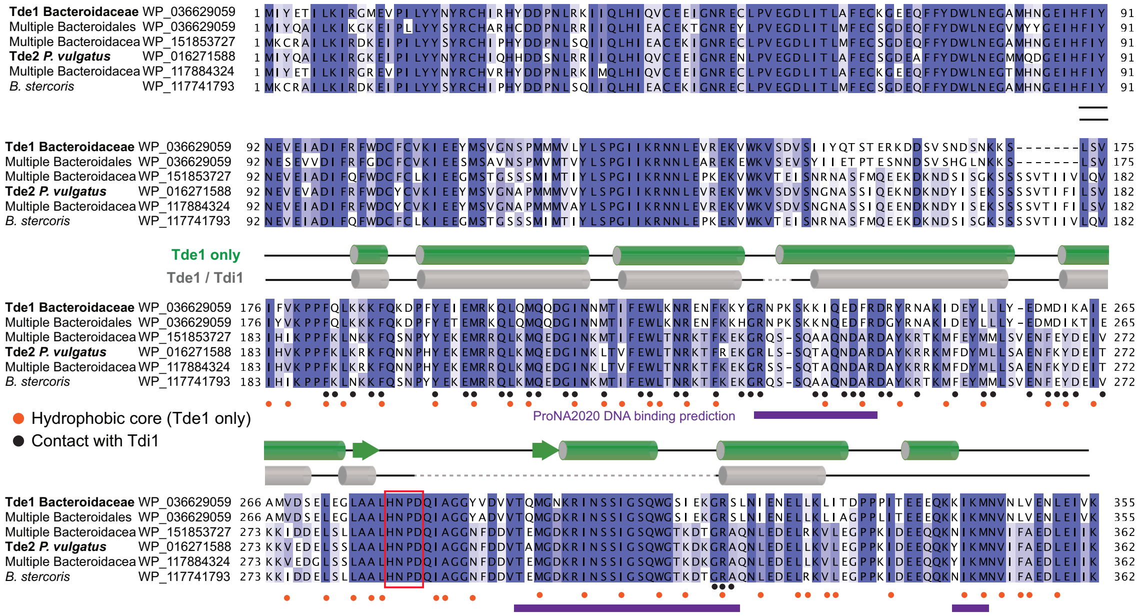

Fig. S5. *Immunity proteins disrupt the active and DNA-binding sites of Ntox15 domains by forming extensive contacts with hydrophobic core residues.* Hcp-Ntox15 domains fusion proteins from several Bacteroidota are aligned. Secondary structural elements of Tde1^{tox} alone are mapped in green, and hydrophobic core residues indicated with orange dots. Secondary structure (gray) and Tdi1 contacts (black dots) are also mapped for the Tde1^{tox} / Tdi1 structure. The largest predicted DNA binding site (purple, ProNA2020 (15)) spans β2 and α6 in the Tde1^{tox} only structure and is disordered in the complex with Tdi1 (dashed lines).

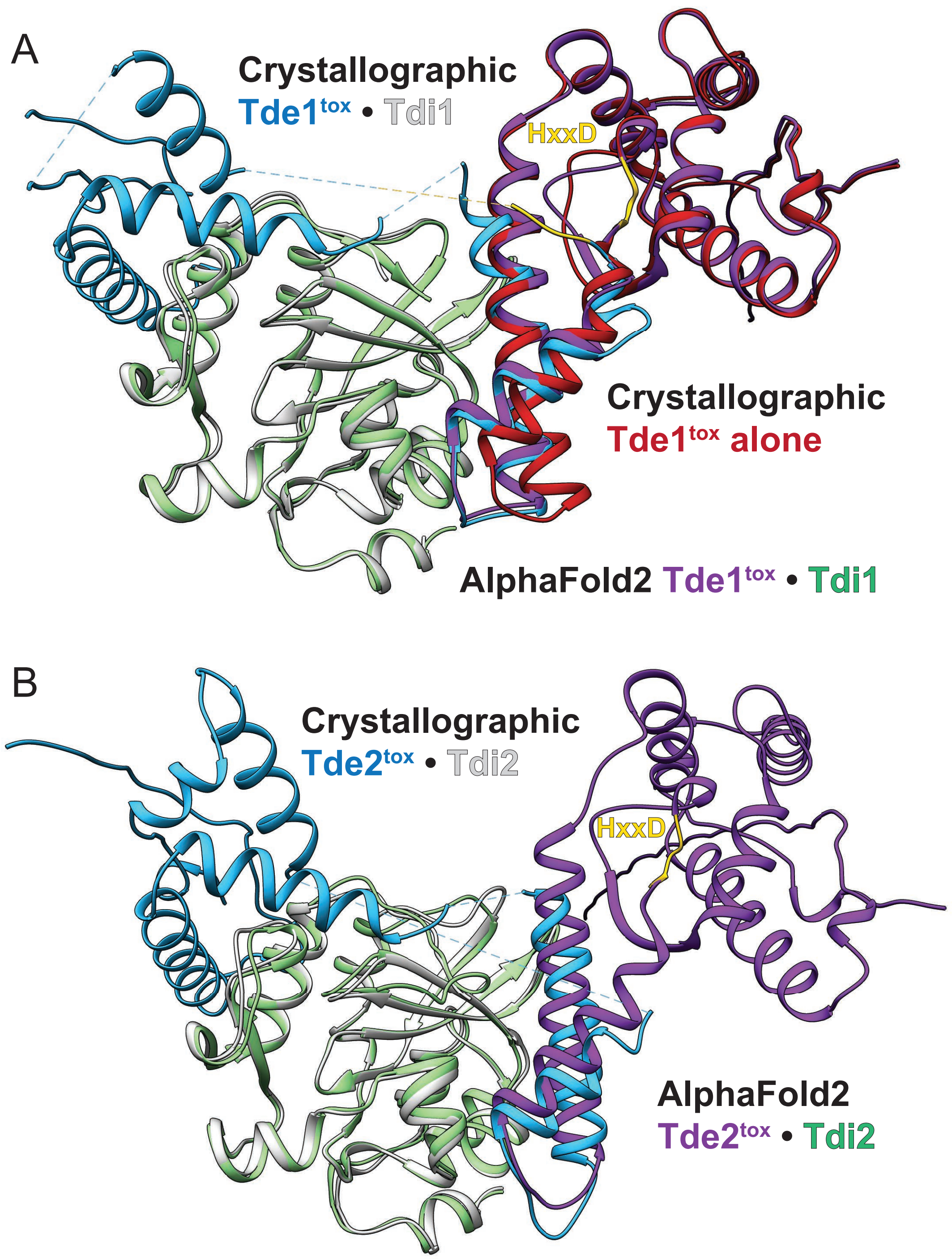

Fig. S6. *Current neural network-based complex modelling does not accurately predict Tde^{tox} / Tdi structures.* A) Crystal structures of Tde1^{tox} / Tdi1 and Tde1^{tox} alone are superimposed with a sequence-only based Tde1^{tox} / Tdi1 prediction using AlphaFold2-multimer. B) Although an experimental structure of Tde2^{tox} alone is not available, the crystal structure of Tde2^{tox} / Tdi1 and a sequence-only based AlphaFold2-multimer are superimposed. Both predictions reflect docking of the individual Ntox15 domain and immunity folds at the α4-α5 interface. The Tde^{tox} conformational changes and secondary interface are not predicted.

	Tde1^{tox} native	Tde2^{tox} / Tdi2 SAD peak	Tde1^{tox} / Tdi1 native
PDB accession code	8FZY	8FZZ	8G0K
**Data Collection**			
Space group	$P2_12_12_1$	$P2_12_12_1$	$C222_1$
Cell dimension			
a, b, c (Å)	86.5, 149.9, 176.5	54.3, 84.8, 175.9	109.8, 112.3, 149.1
α, β, γ (°)	90, 90, 90	90, 90, 90	90, 90, 90
Wavelength (Å)	1.0000	0.9795	1.0000
Resolution (Å)	48.6 - 2.9 (3.24 - 2.90)	39.1 – 2.7 (2.72 – 2.68)	35.0 – 2.2 (2.22 – 2.20)
No. unique reflections	51603 (14277)	42063 (601)	46798 (1159)
R_{merge}	0.15 (1.1)	0.16 (1.2)	0.10 (1.11)
$I/\sigma I$	13.9 (2.1)	19.6 (2.8)	20.6 (2.0)
Completeness (%)			
Total	100 (100)	92.1 (85.2)	99.2 (100)
Anomalous		92.0	
Redundancy	6.6 (6.8)	13.5 (10.7)	10.8 (10.3)
Wilson B-factor (Å^2)	61.5	27.6	34.2
**Refinement**			
Resolution (Å)	48.6 - 2.90 (2.97 - 2.90)	39.0 - 2.68 (2.72 - 2.68)	34.8 - 2.20 (2.25 - 2.20)
No. reflections	51580 (5065)	40563 (1319)	46670 (3022)
Rwork/Rfree (%)	20.2 / 25.8 (31.7 / 40.8)	18.5 / 25.1 (25.6 / 36.1)	20.9 / 26.0 (24.7 / 36.4)
No. atoms	11894	5610	5607
Protein	11687	5609	5257
Ligand/ion	80	0	0
Water	127	1	350
B-factors (Å^2)			
Protein	57.8	33.9	41.9
Ligand/ion	74.5	N/A	N/A
Water	50.3	23.9	43.4
rmsd			
Bond lengths (Å)	0.006	0.008	0.013
Bond angles (°)	0.789	0.930	1.168
Missing residues			
chain A	1-12	1-11, 32-37, 76-79, 123-158, 200-201	1-12, 70-72, 122-153, 168-179, 195
chain B	1-12, 170-172, 196	197-202	1
chain C	1-12, 196	1-13, 76-79, 119-158, 200-201	1-16, 49-56, 122-153, 161-186, 195
chain D	1-12, 196	197-202	1, 195
chain E	1-12, 196		
chain F	1-12, 196		
chain G	1-13, 129-131, 168-182,196		
chain H	1-12, 196		

[a]Values in parentheses are for the highest resolution shell

Table S1. *Crystallographic data*

PDB ID	PubMed ID	Effector	Immunit y	Organism
4NOO	24699653	VgrG3C	TsiV3	*Vibrio cholerae* O1 biovar El Tor str. N16961
6B12	29237732	Tne2	Tni2	*Pseudomonas protegens* Pf-5
4HFK	23288853	Tae4	Tai4	*Enterobacter cloacae* subsp. cloacae ATCC 13047
4JUR	24023903	Tae4	Tai4	*Salmonella enterica* subsp. enterica serovar Typhimurium str. LT2
4NSO	24751834	VgrG3	TsaB	*Vibrio cholerae* O1 biovar El Tor str. N16961
4KT3	23878199	Tge2	Tgi2	*Pseudomonas protegens* Pf-5
6IJF	30511676	Tae4	Tai4	*Agrobacterium tumefaciens*
6OX6	31695193	Tas1	imm.	*Pseudomonas aeruginosa*
4ZV0	36456113	Tse6	Tsi6	*Pseudomonas aeruginosa* PAO1
4BI8	24311588	Ssp1	Rap1a	*Serratia marcescens*
3VPJ	22700987	Tse1	Tsi1	*Pseudomonas aeruginosa* PAO1
3WA5	24100309	Tse3	Tsi3	*Pseudomonas aeruginosa* PAO1
4HZB	23730712	Tae3	Tai3	*Ralstonia pickettii* 12D
4LUQ	24025333	Tse3	Tsi3	*Pseudomonas aeruginosa* PAO1
4EQA	22931054	Tse1	Tsi1	*Pseudomonas aeruginosa* PAO1
6U08	32641830	DddA	DddI	*Burkholderia cenocepacia*
4J30	23826277	Tae4	Tai4	*Salmonella enterica* subsp. enterica serovar Typhimurium str. LT2
7DYM	34371041	TseT	TsiT	*Pseudomonas aeruginosa* PAO1
7JTU	33448264	SsdA	SsdB	*Pseudomonas syringae*
4R1D	25478841	Tle4	Tli4	*Pseudomonas aeruginosa* PAO1
4M5F	24724564	Tse3	Tsi3	*Pseudomonas aeruginosa* PAO1
4N88	24724564	Tse3	Tsi3	*Pseudomonas aeruginosa* PAO1
8BD1	36476863	Rhs	imm.	*Vibrio parahaemolyticus*
6DRE	30343895	ADPR	imm.	*Serratia proteamaculans* 568
5AKO	26749446	Tse2	Tsi2	*Pseudomonas aeruginosa*
7UBZ	N/A	Tle	imm.	*Enterobacter cloacae*
5T86	N/A	CdiA	CdiI	*Escherichia coli*
6VEK	N/A	CdiA	CdiI	*Escherichia coli* 3006
5HKQ	29923643	CdiA	CdiI	*Escherichia coli*
5E3E	28398546	CdiA	CdiI	*Yersinia kristensenii* ATCC 33638
5T87	28960539	CdiA	CdiI	*Cupriavidus taiwanensis* LMG 19424
6CP9	31515004	CdiA	CdiI	*Klebsiella pneumoniae* 342
5I4R	28973472	CdiA	CdiI	*Escherichia coli* NC101
4G6V	23236156	CdiA	CdiI	*Burkholderia pseudomallei* 1026a
4G6U	23236156	CdiA	CdiI	*Escherichia coli* O157:H7
6CP8	31515004	CdiA	CdiI	*Escherichia coli*
4ZQU	26449640	CdiA	CdiI	*Yersinia pseudotuberculosis* YPIII
4NTQ	24657090	CdiA	CdiI	*Enterobacter cloacae* subsp. cloacae ATCC 13047
5J5V	27531961	CdiA	CdiI	*Escherichia coli* O157:H7
7M5F	N/A	CdiA	CdiI	*Serratia marcescens*
5T87	28960539	CdiA	CdiI	*Cupriavidus taiwanensis* LMG 19424
1TFO	15336558	colicin D	imm.	*Escherichia coli*
5EW5	27402794	colicin E9	imm.	*Escherichia coli*
1MZ8	12441392	colicin E7	imm.	*Escherichia coli*
2K5X	18980319	colicin E9	imm.	*Escherichia coli*
2JBG	17368670	colicin E7	imm.	*Escherichia coli* str. K-12 substr. W3110
1E44	10986462	colicin E3	imm.	*Escherichia coli*
2DFX	17099236	colicin E5	imm.	*Escherichia coli*
1BXI	N/A	colicin E9	imm.	*Escherichia coli*
1UJZ	15034550	colicin E7	imm.	*Escherichia coli*
7CEI	10368275	colicin E7	imm.	*Escherichia coli* str. K-12 substr. W3110
3U43	22306467	colicin E2	imm.	*Escherichia coli*
1V74	15014439	colicin D	imm.	*Escherichia coli*
2FHZ	16524591	colicin E5	imm.	*Escherichia coli*
1EMV	10966813	colicin E9	imm.	*Escherichia coli*

1JCH	11741540	colicin E3	imm.	*Escherichia coli* str. K-12 substr. W3110
4UHP	26215615	pyocin AP41	imm.	*Pseudomonas aeruginosa* PAO1
4QKO	N/A	pyocin S2	imm.	*Pseudomonas aeruginosa* PAO1
6W0V	32817098	pyocin S8	imm.	*Pseudomonas aeruginosa*
4QLP	26237511	TNT	IFT	*Mycobacterium tuberculosis* H37Rv
6ZN8	33096014	vapD	vapX	*Haemophilus influenzae* 86-028NP
7RT7	N/A	RhsP2	RhsI2	*Pseudomonas aeruginosa* UCBPP-PA14
8BD1	N/A	Rhs	imm.	*Vibrio parahaemolyticus*
7ZHM	34255843	Rhs1	TriTu	*Salmonella enterica* subsp. enterica serovar Typhimurium
3PNT	21300288	NAD glyc	imm.	*Streptococcus pyogenes*
8GUO	36307446	EsaD	EsaG	*Staphylococcus aureus* subsp. aureus NCTC 8325

Table S2. *Polymorphic toxin structures analyzed for solvation energy calculations*

Reagent	Source	Identifier
**Chemicals, peptides, recombinant proteins**		
Phusion polymerase	ThermoFisher	F530
Gentamycin	Research Products International	G38000
Tetracycline	Research Products International	T17000
Erythromycin	Fisher Scientific	BP920
Ampicillin	Research Products International	A40040
Anhydrotetracycline	Fisher Scientific	AC233131000
5-fluorodeoxyuracil (FUdR)	Bioworld	40690016
Brain-heart infusion broth	ThermoFisher	250220
Agar	Research Products International	A20030
Agarose	Midsci	MIDSCI-500
Gibson assembly master mix	Invitrogen	A46629
Guanidine HCl	ThermoFisher	24110
Tobacco etch virus protease	Iowa Protein and Crystallography core facility	N/A
Ethylenediaminetetraacetic acid (EDTA)	Research Products International	E57045
Sodium chloride	Research Products International	S23025
Imidazole	Sigma-Aldrich	I202
Dithio-threitol (DTT)	Sigma-Aldrich	D0632
HEPES	Sigma-Aldrich	H3375
Isopropyl β-D-1-thiogalctopyranoside (IPTG)	Research Products International	I56000
Borane dimethylamine complex	Millipore Sigma	180238
Ammonium sulfate	Research Products International	A20510
Sodium acetate	Bio-chem	1005328
Sucrose	Sigma-Aldrich	S9378
Polyethylene glycol (PEG 3350)	Hampton Research	HR2-591
Sodium citrate	Research Products International	S23040
Ethylene glycol	Research Products International	324558
Selenomethionine *E. coli* media	Molecular dimensions	MD12-500
SYPRO orange	Fisher Scientific	S6650
Magnesium chloride hexahydrate	Research Products International	M24000
Zinc chloride	Millipore Sigma	229997
Calcium chloride	Millipore Sigma	C4901
DNA electrophoresis loading dye	Fisher Scientific	AAJ63869AD
**Bacterial strains**		
Escherichia coli EC100D *pir*	Lucigen	ECP09500
Escherichia coli S17-1 λ *pir*	Gift from Dr. Ben Ross	N/A
Escherichia coli BL21	Millipore Sigma	69450
Escherichia coli B834	Millipore Sigma	69041
Escherichia coli DH5α	ThermoFisher	18258012
Phocaeicola vulgatus	ATCC	8482
Bacteroides thetaiotaomicron	Eric Pamer laboratory, University of Chicago	MSK.16.9
Bacteroides dorei	Eric Pamer laboratory, University of Chicago	MSK.16.93
Phocaeicola vulgatus	Eric Pamer laboratory, University of Chicago	MSK.16.2
Bacteroides thetaiotaomicron	Eric Pamer laboratory, University of Chicago	MSK.16.5
Bacteroides xyanlisolvens	Eric Pamer laboratory, University of Chicago	MSK.16.13
Bacteroides uniformis	Eric Pamer laboratory, University of Chicago	MSK.16.7
Phocaeicola vulgatus	Eric Pamer laboratory, University of Chicago	MSK.16.10
Phocaeicola vulgatus MSK 16.10, Δ*tdk*, Δ*tde1*Δ*tdi1*, Δ*tdk*Δ*tde1*Δ*tdi1*	This study	NA
Phocaeicola vulgatus ATCC 8482, Δtdk pNBU2::*tdi1*-VSVG, pNBU2::tdi_{oA}-VSVG, pNBU2:: tdi_{oB}-VSVG	This study	NA
Phocaeicola vulgatus MSK16.2, Δ*tdk*, Δ*tde1*Δ*tdi1*, Δ*tssB*	This study	NA
**Critical commercial assays**		
DNeasy Blood and Tissue kit	Qiagen	69504
Octet BLI biosensors (NTA, streptavidin) and kinetics buffer	Sartorius	18-5101, 18-5019, 18-1105
**Deposited Data**		

X-ray diffraction data	This study	Protein databank IDs 8FZY, 8FZZ, 8G0K
**Recombinant DNA**		
pNBU2_erm_P1T_DP-B1	Addgene	90319
pNBU2_erm_P1T_DP-B1 *tdi1*-VSVG	This study	N/A
pNBU2_erm_P1T_DP-B1 tdi_{oA}-VSVG	This study	N/A
pNBU2_erm_P1T_DP-B1 tdi_{oB}-VSVG	This study	N/A
pLGB30	Addgene	126620
pLGB30 Δ*tdk*, Δ*tde1*Δ*tdi1*	This study	N/A
pETDuet-1	Millipore Sigma, Novagen	71146
pETDuet-1 *tde1* Ntox15 domain + *tdi1*, *tde1* HxxD->AxxA	This study	N/A
pcDNA3.1	Invitrogen	V79020
**Software and algorithms**		
GraphPad Prism 9	GraphPad, La Jolla, CA, USA	https://www.graphpad.com/
HKL2000	HKL Research, Inc.	https://hkl-xray.com/hkl-2000
Coot	(5)	https://www2.mrc-lmb.cam.ac.uk/personal/pemsley/coot/
Phenix	(16)	https://phenix-online.org/
AlphaFold2	(3)	https://colab.research.google.com/github/sokrypton/ColabFold/blob/main/AlphaFold2.ipynb
BLAST	NCBI	https://www.ncbi.nlm.nih.gov/books/NBK279690/
Clustal omega	EMBL-EBI	https://www.ebi.ac.uk/Tools/msa/clustalo/
RStudio	Posit	https://posit.co/
HMMer	(9)	http://hmmer.org/
Metaphlan3	(17)	https://huttenhower.sph.harvard.edu/metaphlan/
Geneious 11	Biomatters	https://www.geneious.com/
Chimera	(18)	https://www.cgl.ucsf.edu/chimera/
Adobe Illustrator	Adobe	https://www.adobe.com/products/illustrator.html

Table S3. *Materials, deposited data and software.*

SI References

1. Tan K, Kim Y, Hatzos-Skintges C, Chang C, Cuff M, Chhor G, Osipiuk J, Michalska K, Nocek B, An H, Babnigg G, Bigelow L, Joachimiak G, Li H, Mack J, Makowska-Grzyska M, Maltseva N, Mulligan R, Tesar C, Zhou M, Joachimiak A. 2014. Salvage of failed protein targets by reductive alkylation. Methods Mol Biol 1140:189-200.
2. Otwinowski Z, Minor W. 1997. Processing X-ray Diffraction Data Collected in Oscillation Mode, p 307-326. *In* Carter Jr C (ed), Methods in Enzymology, vol 276. Academic Press, New York.
3. Mirdita M, Schütze K, Moriwaki Y, Heo L, Ovchinnikov S, Steinegger M. 2021. ColabFold - Making protein folding accessible to all. bioRxiv doi:10.1101/2021.08.15.456425:2021.08.15.456425.
4. Adams PD, Afonine PV, Bunkoczi G, Chen VB, Echols N, Headd JJ, Hung LW, Jain S, Kapral GJ, Grosse Kunstleve RW, McCoy AJ, Moriarty NW, Oeffner RD, Read RJ, Richardson DC, Richardson JS, Terwilliger TC, Zwart PH. 2011. The Phenix software for automated determination of macromolecular structures. Methods 55:94-106.
5. Casañal A, Lohkamp B, Emsley P. 2020. Current developments in Coot for macromolecular model building of Electron Cryo-microscopy and Crystallographic Data. Protein Sci 29:1069-1078.
6. Painter J, Merritt EA. 2006. Optimal description of a protein structure in terms of multiple groups undergoing TLS motion. Acta Crystallogr D Biol Crystallogr 62:439-50.
7. Lim B, Zimmermann M, Barry NA, Goodman AL. 2017. Engineered Regulatory Systems Modulate Gene Expression of Human Commensals in the Gut. Cell 169:547-558 e15.
8. Oliveira F, Giorgobiani E, Guimaraes-Costa AB, Abdeladhim M, Oristian J, Tskhvaradze L, Tsertsvadze N, Zakalashvili M, Valenzuela JG, Kamhawi S. 2020. Immunity to vector saliva is compromised by short sand fly seasons in endemic regions with temperate climates. Sci Rep 10:7990.
9. Potter SC, Luciani A, Eddy SR, Park Y, Lopez R, Finn RD. 2018. HMMER web server: 2018 update. Nucleic Acids Res 46:W200-W204.
10. Integrative HMPRNC. 2014. The Integrative Human Microbiome Project: dynamic analysis of microbiome-host omics profiles during periods of human health and disease. Cell Host Microbe 16:276-89.
11. Segata N, Waldron L, Ballarini A, Narasimhan V, Jousson O, Huttenhower C. 2012. Metagenomic microbial community profiling using unique clade-specific marker genes. Nat Methods 9:811-4.
12. Sorbara MT, Littmann ER, Fontana E, Moody TU, Kohout CE, Gjonbalaj M, Eaton V, Seok R, Leiner IM, Pamer EG. 2020. Functional and Genomic Variation between Human-Derived Isolates of Lachnospiraceae Reveals Inter- and Intra-Species Diversity. Cell Host Microbe 28:134-146 e4.
13. Forster SC, Kumar N, Anonye BO, Almeida A, Viciani E, Stares MD, Dunn M, Mkandawire TT, Zhu A, Shao Y, Pike LJ, Louie T, Browne HP, Mitchell AL, Neville BA, Finn RD, Lawley TD. 2019. A human gut bacterial genome and culture collection for improved metagenomic analyses. Nat Biotechnol 37:186-192.
14. Sievers F, Higgins DG. 2014. Clustal omega. Curr Protoc Bioinformatics 48:3.13.1-16.
15. Qiu J, Bernhofer M, Heinzinger M, Kemper S, Norambuena T, Melo F, Rost B. 2020. ProNA2020 predicts protein-DNA, protein-RNA, and protein-protein binding proteins and residues from sequence. J Mol Biol 432:2428-2443.
16. Liebschner D, Afonine PV, Baker ML, Bunkoczi G, Chen VB, Croll TI, Hintze B, Hung LW, Jain S, McCoy AJ, Moriarty NW, Oeffner RD, Poon BK, Prisant MG, Read RJ, Richardson JS, Richardson DC, Sammito MD, Sobolev OV, Stockwell DH, Terwilliger TC, Urzhumtsev AG, Videau LL, Williams CJ, Adams PD. 2019. Macromolecular structure determination using X-rays, neutrons and electrons: recent developments in Phenix. Acta Crystallogr D Struct Biol 75:861-877.
17. Truong DT, Tett A, Pasolli E, Huttenhower C, Segata N. 2017. Microbial strain-level population structure and genetic diversity from metagenomes. Genome Res 27:626-638.

18. Pettersen EF, Goddard TD, Huang CC, Couch GS, Greenblatt DM, Meng EC, Ferrin TE. 2004. UCSF Chimera--a visualization system for exploratory research and analysis. J Comput Chem 25:1605-12.
